# Dietary Oxidative Distress: A Review of Nutritional Challenges as Models for Poultry, Swine and Fish

**DOI:** 10.3390/antiox10040525

**Published:** 2021-03-27

**Authors:** Elodie Bacou, Carrie Walk, Sebastien Rider, Gilberto Litta, Estefania Perez-Calvo

**Affiliations:** 1 DSM Nutritional Products, Animal Nutrition and Health, F-68128 Village-Neuf, France; sebastien.rider@dsm.com (S.R.); estefania.perez-calvo@dsm.com (E.P.-C.); 2 DSM Nutritional Products, Animal Nutrition and Health, Wurmisweg 576, 4303 Kaiseraugst, Switzerland; carrie.walk@dsm.com (C.W.); gilberto.litta@dsm.com (G.L.)

**Keywords:** oxidative distress, challenge models, antioxidants, diet, gastrointestinal, pigs, poultry, fish

## Abstract

The redox system is essential for maintaining cellular homeostasis. When redox homeostasis is disrupted through an increase of reactive oxygen species or a decrease of antioxidants, oxidative distress occurs resulting in multiple tissue and systemic responses and damage. Poultry, swine and fish, raised in commercial conditions, are exposed to different stressors that can affect their productivity. Some dietary stressors can generate oxidative distress and alter the health status and subsequent productive performance of commercial farm animals. For several years, researchers used different dietary stressors to describe the multiple and detrimental effects of oxidative distress in animals. Some of these dietary challenge models, including oxidized fats and oils, exposure to excess heavy metals, soybean meal, protein or amino acids, and feeding diets contaminated with mycotoxins are discussed in this review. A better understanding of the oxidative distress mechanisms associated with dietary stressors allows for improved understanding and evaluation of feed additives as mitigators of oxidative distress.

## 1. Introduction

Redox homeostasis is an essential mechanism for aerobic organisms (bacteria, plants, animals and humans). Under physiological conditions, cells maintain redox homeostasis through the production of oxidants, reactive oxygen species (ROS) and their elimination by antioxidant system. ROS include free radical species such as superoxide, hydroxyl radical and singlet oxygen, along with non-radical species such as hydrogen peroxide (H_2_O_2_). All these species are involved in different cellular processes such as proliferation, differentiation, and regulation of the immune system [1]. Redox homeostasis can be disrupted through an increase of ROS.

In 1985, Sies defined “oxidative stress” as an “imbalance between oxidants and antioxidants in favor of oxidants” [2]. The definition of oxidative stress has been recently updated. Two aspects of oxidative stress were recognized (1) a minimum amount of oxidant production essential for life process through redox signaling, called oxidative eustress and (2) an overexposure to oxidants resulting in non-specific oxidation of biomolecules and disruption of redox signaling termed oxidative distress [3].

In farming conditions, animals such as poultry, swine and fish are exposed to several environmental, technological, chemical, and nutritional stressors resulting in potential oxidative distress. Nutritional stressors present an important challenge to production animals. Indeed, nutritional stress leads to decreased productive and reproductive performance as well as impairments in health, which can result in economic losses for farmers [4,5,6]. In the animal, oxidative distress is mitigated by endogenous antioxidants which act to decrease the production of ROS [4]. For these reasons, it is important to better understand the mechanisms of oxidative distress in these animals and the modes of action of the endogenous antioxidant systems.

In this review, we will discuss (1) the function of ROS in conditions of oxidative eustress and distress, (2) the mechanisms of the endogenous antioxidant system, and (3) the impact of nutritional challenge models on production parameters and biomarkers of oxidative distress in poultry, swine, and fish.

## 2. Redox Signaling

ROS are important for the maintenance of cellular homeostasis and production comes from numerous different sources. ROS have regulatory functions at the transcriptional level, but over production of ROS can induce cellular damage.

### 2.1. Source of ROS

Two main sources of ROS include the electron transport chain in the mitochondria and the NADPH oxidases located in the cell membrane. The mitochondria are the major site for ROS production thought the electron transport chain. The mitochondrial electron transport chain consists of four complexes (I, II, III, IV), ATP synthase, coenzyme Q, and cytochrome *c*. Electrons come from the reducing of NADH (complex I) or FADH2 (complex II) and pass through the mitochondrial electron transport chain to reduce oxygen into water at the ATP synthase complex. The production of ROS, mainly superoxide, takes place during the leakage of electrons, predominantly from complexes I and III [7]. Complex I releases superoxide anion into mitochondrial matrix while complex III releases it into both sides of the inner mitochondrial membrane [8].

For a long time, it was thought that NADPH oxidase was only expressed on phagocyte cells to generate a high amount of ROS, allowing pathogen degradation in a process called “oxidative burst” or “respiratory burst”. In recent years, it has been reported that low levels of ROS are produced by NADPH oxidase in non-phagocyte cells [9]. NADPH oxidases are transmembrane enzymes and seven different homologues have been identified in the mammalian genome: NOX1 to NOX5, DUOX1, and DUOX2. All of the NOX family isoforms have a conserved common structure [10]. The main function of the NOX enzymes is the production of ROS, but the types of ROS generated differ while NOX-1, -2, -3 and -5 generate mainly superoxide anions, NOX-4, DUOX1 and DUOX2 produce mainly H_2_O_2_ [11].

### 2.2. ROS Cell Signaling

At low to modest levels, ROS (primarily H_2_O_2_), act as second messengers of signal transduction in different intracellular functions, such as cell proliferation, apoptosis, and cell survival and differentiation. The transcription factor, NF-κB (nuclear factor kappa-light-chain-enhancer of activated B cells) is involved in different cell mechanisms like inflammation, cell survival and proliferation. The NF-κB complex protein consists of Rel, RelA (p65), RelB, p50 and p52 and its activity is regulated by inhibitory proteins called IκBs [12]. The regulation of NF-κB can be modulated by ROS both at the nuclear and cytoplasmic level. At the nuclear level, oxidation of the redox-sensitive site Cys62 of the p50 subunit inhibits its ability to bind DNA [13]. Oxidation of p50 is reversible and DNA binding can be restored through reduction by thioredoxin [14]. At the cytoplasmic level, H_2_O_2_ can activate NF-κB activity through direct phosphorylation of IKK (IkB kinase). Indeed, H_2_O_2_ affects the phosphorylation of IκBα on Tyr42 inducing the degradation of IκBα and activation of NF-κB pathways [15]. Epithelial NF-κB activation occurs in response to enteric pathogens, Toll-like receptor signaling and pro-inflammatory cytokines [16]. Activation of NF-κB also has an effect on tight junction mediated epithelial permeability [17].

Nrf2 (Nuclear factor erythroid-2 related factor 2) is considered as a sentinel of oxidative stress and protects the body by making it more resistant to an increase in ROS. Nrf2 generally binds to Kelch-like ECH associated protein 1 (Keap1) in the cytoplasm. Under normal conditions, Nrf2 is targeted by Keap1, which promotes Nrf2 proteasomal degradation via interactions with a ubiquitin ligase [18]. When the intracellular level of ROS increases, the key cysteine residues (Cys273, Cys288 and Cys151) are oxidized. This reaction decreases the ability of Keap1 to bind Nrf2 allowing the release of Nrf2 to Keap1. Then, Nrf2 is phosphorylated via the activation of kinase and translocated to the nucleus. In the nucleus Nrf2 dimerize with the small Musculoaponeurotic fibrosarcoma (Maf) proteins and bind to ARE (antioxidant response element) of phase II genes [19]. The Nrf2-ARE complex controls the expression of important proteins and enzymes involved in redox balance, such as thioredoxin 1, thioredoxin reductase 1, sulfiredoxin 1, NADPH-generating enzymes, and the glutathione-based system [20].

### 2.3. Indirect Methods for Measuring Oxidative Distress

In “physiological” conditions, ROS production is low but essential for maintaining cellular homeostasis. Under conditions of oxidative distress, high ROS production induces cell damage, such as lipid peroxidation and protein and DNA oxidation. These oxidative damages can be measured as an indirect method to determine the level of oxidative distress. ROS can be also analyzed directly by electro spin resistance/trapping or fluorescent probes. However, due to the short half-life of ROS, it is difficult to measure it in the laboratory [21]. Therefore, only indirect methods will be described below.

Lipid peroxidation is the process in which ROS predominantly attack polyunsaturated fatty acids (PUFA) present in the cell membrane, resulting in membrane disruption and cell death [22]. Some lipid peroxidation by-products, like malondialdehyde (MDA), thiobarbituric acid reactive substances (TBARS), isoprostanes (ISP) and 4-hydroxyalkenals (4-HNE) are used as biomarkers of lipid peroxidation [23]. The MDA and TBARS assays are the most used biomarkers to determine lipid peroxidation. However, their use is controversial, indeed, MDA is not representative of all aldehydes produced during lipid peroxidation and TBARS are not specific to aldehydes. ISPs are currently seen as the best marker of lipid peroxidation because they are specific end products of PUFA peroxidation [24].

As lipids, proteins can be attacked by free radicals resulting in amino-acid modifications. These modifications induce the production of carbonyl groups on protein side chains and a loss of protein functions [25]. Different assays are available for the detection of protein carbonyls, such as spectrophotometric, HPLC, ELISA and immunoblotting methods [26].

DNA can also be attacked by free radicals and mainly hydroxyl radicals. A well-known marker of DNA oxidation is the 8-hydro-2′-deoxyguanosine (8-OHdG) analyzed with different methods like ELISA and HPLC. This marker is also measurable in the urine, a non-invasive matrix [27]. Another assay that can be used to analyze DNA damage is the comet assay measuring single or double DNA breaks depending on the assay variant [28].

## 3. Endogenous Antioxidant System

The endogenous antioxidant system protects the body by modulating the free radical reactions to maintain redox homeostasis. These antioxidants are enzymatic or non-enzymatic.

### 3.1. Enzymatic Antioxidants

The endogenous antioxidant enzyme system is composed of superoxide dismutase (SOD), catalase (CAT), and glutathione peroxidase (GPX). These enzymes are mainly involved in protecting the cell against significant increases of ROS by breaking down and removing free radicals.

Three distinct isoforms of SOD have been identified with the same mode of action [29,30]. The catalysis of superoxide radicals occurs at the reactive metal site in two steps. First, one superoxide radical interacts with the metal cation to reduce it and generates an oxygen molecule. Then, a second superoxide radical oxidizes the reduced metal cation with the release of H_2_O_2_ [31].

CAT is a homo-tetramer (240 kDa) found in the peroxisomes of cells and is one of the most well characterized antioxidant enzymes. CAT possesses four iron-containing heme groups at the active site. The catalytic reaction occurs in two steps whereby catalase heme Fe^3+^ reduces one molecule of H_2_O_2_ to water, generating a covalent Fe^4+^O oxyferryl species and a porphyrin cation radical. First, one H_2_O_2_ molecule acts as an oxidant by oxidizing the CAT to compound I. Then, a second molecule of H_2_O_2_, acting as a reducing agent, reduces compound I back to the resting state and generates water and molecular oxygen. However, the CAT can act as peroxidase. Indeed, after compound I formation, it can be reduced by a single electron to form a stable compound II. This compound is finally reduced back to ferric peroxidase by a second one-electron reduction. To prevent formation of inactive compound II from compound I, NADPH can tightly bind each CAT subunit [32,33]. The affinity of CAT for H_2_O_2_ is very low (Km > 10 mM), and at “physiological” H_2_O_2_ concentrations, peroxidatic activity seems to predominate. While at high concentrations of H_2_O_2_, catalytic reactions predominate [34].

Eight isoforms of GPXs have been identified (GPX1-8) but only the functions of four GPXs are well characterized (GPX1-4). The different GPXs are found in different tissues and subcellular locations [35]. These GPXs, containing selenocysteine residues in their active sites, can reduce the H_2_O_2_ to water and lipid hydroperoxides to corresponding stable alcohols [36]. However, unlike CAT, this reaction requires glutathione as a substrate. GPXs oxidize glutathione into oxidized glutathione, also called glutathione disulfide (GSSG).

### 3.2. Non-Enzymatic Antioxidants

The non-enzymatic antioxidants do not act directly on free radicals but they interrupt their chain reactions. The endogenous non-enzymatic antioxidant system is composed of lot of compounds such as glutathione, vitamins, and alpha-lipoic acid.

Glutathione exists in two forms: reduced (GSH) and oxidized (GSSG). Under normal conditions the predominant form of glutathione is the GSH (almost 98%). Glutathione is used as a substrate for GPX during the reduction of H_2_O_2_ [37]. Furthermore, glutathione is also involved in a process called “protein S-glutathionylation” modulating protein functions [38]. This mechanism represents a formation of a disulfide bond between a protein thiol and GSH. During oxidative distress, ROS oxidize sulfenates (R-SOH) to sulfinates (R-SO_2_H) and sulfonates (R-SO_3_H), which is an irreversible mechanism resulting in permanent protein structure and functional damage. GSH protects protein thiols against this irreversible oxidation. These modifications, induced by GSH, can be reversed by the enzymes thioredoxin or glutaredoxin. Protein S-glutathionylation is also an important mechanism for maintaining the redox environment in the cell (ratio GSSG/GSH) and for preventing the loss of GSH under oxidative distress conditions [39,40].

The potent antioxidant activity of vitamins, mainly E, C, A and B_2_, are well known. The vitamins are described following their antioxidant potency. Even if they are not synthetized by the body, the dietary supply is stored to deal with oxidative distress conditions. Vitamin E is an efficient fat-soluble antioxidant. It is well-known that among all vitamin E isomers, the best antioxidant is the α-tocopherol [41]. Vitamin E acts as “chain breaker” able to interrupt the lipid peroxidation process [42]. Vitamin C, also known as ascorbic acid, is a potent water-soluble antioxidant able to scavenge oxidants and to regenerate other antioxidants, such as vitamin E. Indeed, vitamin C can restore α-tocopherol function with an electron donation. The oxidized form of vitamin C (dehydroascorbate) can be reduced back into ascorbic acid in the presence of GSH [43]. Vitamin A, as vitamin E, is a fat-soluble antioxidant. Among the two different families of vitamin A, only the carotenoids have a direct antioxidant function [44]. The carotenoids act as a singlet oxygen scavenger. Vitamin B_2_ (also known as riboflavin) is a water-soluble vitamin and the most effective antioxidant in the blood. This vitamin is known for protecting cells against lipid peroxidation [45].

Alpha-lipoic acid is a substance synthetized in the mitochondria from octanoic acid and cysteine by the action of lipoic acid synthase. Similar to glutathione, alpha-lipoic acid is present in two forms: oxidized (disulfide, alpha-lipoic acid, ALA) and reduced (dihydrolipoic acid, DHLA). Both forms can scavenge ROS and the reduced form can regenerate endogenous antioxidants, such as glutathione and vitamin C [46]. Among the different ROS, the hydroxyl radical and the peroxide radical are scavenged by both ALA and DHLA, while superoxide is scavenged only by DHLA. However, for H_2_O_2_, neither forms are active [47,48]. Once the antioxidant is oxidized, it is not able to scavenge ROS. DHLA is a potent agent reducing some oxidized forms of dehydroascorbate and GSSG. These mechanisms are characterized by an increase of ascorbate and GSH in the cell which can contribute to vitamin E regeneration from radical form [49].

Due to cell damage associated with an over-production of ROS, the endogenous antioxidant system works to maintain redox homeostasis. This system is composed of enzymatic antioxidants, such as CAT, SOD and GPX, which act to break down or remove free radicals and non-enzymatic antioxidants including fat soluble vitamins A and E, water soluble vitamins C and B_2_, glutathione and alpha-lipoic acid.

## 4. Dietary Oxidative Distress Challenges Models in Poultry, Swine and Fish

Numerous dietary interventions based on optimal supplementation of antioxidative vitamins (E, A, C and B_2_), micronutrients (Se, Cu and Zn) and feed additives are available to mitigate oxidative distress in poultry, swine [50] and fish [51,52]. However, due to the multiple factors that cause oxidative distress in farm animals, such as infectious diseases, management stressors (temperature, density, or transport) and dietary manipulations, it is difficult to show a consistent dietary antioxidant intervention response. Over the years, researchers have used many different in vivo challenge models to generate oxidative distress [53]. The models allow researchers to evaluate the efficacy of feed additives to mitigate oxidative distress via measurements of the antioxidant potential of the gut, and/or the interactions between dietary oxidants and antioxidants. This review will focus on gastrointestinal oxidative distress caused by dietary challenges relevant to commercial animal production conditions, and the oxidative distress response of poultry, swine and fish.

This section will focus on three different dietary stressors that might be common and can affect poultry and pig production, including: (1) harmful feed ingredients, such as peroxidized lipids [54], (2) nutrient imbalances, such as heavy metals and amino acid deficiencies, and (3) mycotoxins, which induce the generation of cellular free radicals resulting in redox imbalances at the gut level. In turn, these stressors will affect the health status and productivity of chickens and pigs [5]. Similarly, a myriad of factors are documented to induce oxidative distress in both wild and cultured fish. These may be broadly divided into four groups: (1) chemotoxicity-induced, including contaminants such as heavy metals, petroleum derivatives, herbicides and pesticides, (2) environmentally induced, by extremes of water temperature, salinity, oxygen, or ozonation, (3) aquaculture-specific, for example fish transport, handling, crowding, fasting or nitrogenous wastes, notably ammonia [52,55] and (4) diet-induced, that is arising from inadequate consumption of essential nutrients including vitamins, essential amino acids or elminerals, or postprandial oxidative distress resulting from the consumption of physiologically challenging macro-ingredients [56]. The necessity of dietary nutrients, such as vitamins, in the maintenance of redox balance and gastrointestinal health and function has been well-studied [51,57,58]. “Postprandial oxidative distress” describes the susceptibility of an organism towards oxidative damage after consumption of a physiologically challenging ingredient. Due to increasing use of least-cost formulation strategies, commercial fish diets will rely on an increasing variety of macro-ingredients and a diversity of functional additives [56]. High SBM inclusion and oxidized fish oils may be used as production-relevant models for the induction postprandial oxidative distress in vivo, against which the efficacy of novel functional additives may be tested.

### 4.1. Oxidized Fat

Supplemental fats and oils are commonly added to livestock diets as an efficient source of energy and essential source of fatty acids. Dietary fats and oils also increase palatability, improve pellet quality, reduce dustiness, and improve absorption of fat-soluble vitamins [54,59]. However, lipid peroxidation is a complex and dynamic process that can happen frequently during feed manufacturing (pelleting and extrusion) and storage. The oxidation of lipids results in a variety of degradation products that are differentially absorbed and metabolized. For example, the oxidation of essential fatty acids in fish oils results in the loss of their nutritional benefits, leading to harmful effects arising from primary and secondary oxidation products [60]. During the process of lipid auto-oxidation in feedstuffs, the chemical degradation products that are formed include free radicals, peroxides, aldehydes and ketones. In the presence of oxygen, the initial stages of fatty acid oxidation occurs slowly and is initiated by the abstraction of a hydrogen atom from a methylene group (CH2) at susceptible unsaturated carbon-to-carbon bonds. The resulting primary oxidation products are highly reactive hydroperoxides. Subsequently, shorter chain secondary oxidation products including aldehydes, ketones, alcohols and alkanes are produced in a chain-reaction propagated by the concurrent production of peroxyl and hydroxyl radicals [61]. The primary and secondary degradation products of oxidized lipids react with other dietary ingredients including vitamins, proteins and other lipids, which can diminish their nutritional value. The rate of lipid oxidation in aerobic conditions is dependent upon many factors including the lipid to protein ratio, the composition of fatty acids, the presence of one or more reducing agents [61] and the presence of transition metals, notably iron [62].

Large variation exists in the quality of oils and fats fed to livestock. A recent survey indicates that the peroxide value (PV, a simple and rapid analysis used to determine lipid quality) can vary from 0.1 to 181 mEq/kg lipid [54]. Lipid peroxidation depends on the degree of lipid saturation, temperature, exposure to oxygen, light, transition metals, undissociated salts, water, and other non-lipid compounds [63]. By-products of lipid peroxidation can be damaging both through the formation of reactive radicals and through the direct covalent interactions between some peroxidation products, and biological molecules [64,65,66]. In that regard, consumption of peroxidized lipids by animals with inadequate supplies of endogenous antioxidants may develop metabolic oxidative distress. Several studies evaluating peroxidized lipids in the diets of different animal species, has resulted in losses of fat-soluble vitamins, growth performance (decreased feed intake, growth rate and gain efficiency), reduced energy digestibility, meat quality, increased metabolic oxidative status, inflammatory reactions and an increase in susceptibility to other stressors and diseases that may lead to an increased mortality.

- Poultry

In poultry, the body composition of fat is similar to the composition of fat from the diet [59]. Consumption of oxidized lipids results in lipid peroxidation in the tissues and reduced meat quality characteristics, such as an increase in drip and cooking losses and undesirable changes in meat color and pH [67]. Adding oxidized (often, heat-treated) lipids to the diet has been shown to slow growth rate and increase oxidative damage in poultry (Table 1).

As reported by Engberg et al. (1996), broilers receiving oxidized vegetable oil had a depression in growth rate and the animals had a greater concentration of TBARS and a lower concentration of antioxidants in the in plasma or tissues [68]. Tavarez et al. (2011) reported broilers fed 7 mEq oxidized soy oil/kg diet had significantly reduced final body weight, body weight gain and feed efficiency [69]. Live weight, carcass weight and yellowness of the breast meat were also negatively influenced by oxidized soy oil. Broilers fed 17 mEq oxidized rapeseed and soybean oil blend/kg diet tended to have decreased live weight gain [68] and feeding 12 mEq oxidized soybean oil/kg diet decreased intake and increased feed conversion ratio (FCR), with no impact on gain of young broilers [70]. Laying hens fed oxidized vegetable oil had decreased feed intake, decreased egg production and egg weight and increased FCR [71,72,73]. Feeding broiler chickens an oxidized animal/vegetable fat blend (75 and 150 mEq/kg lipid) from hatch to market age significantly decreased body weight gain and increased FCR but had no impact on carcass yield [74]. In a repeat experiment and using the same method and type of oxidized oil, only feed efficiency was significantly and negatively impacted when broilers were fed 75 and 150 mEq/kg oxidized lipids, with no impact on gain or carcass yield [75]. 

Other authors have found no negative effect of oxidized oil or fat on broiler chicken production parameters. For example, increasing the dietary concentration of oxidized poultry fat from 0, 3 or 6 mEq fat/kg diet or oxidized soybean oil from 1, 2 or 3 mEq oil/kg diet had no impact on body weight or FCR of young broilers [76,78]. Similarly, oxidized poultry fat had no impact on weight gain or carcass and breast meat yield, and significantly improved FCR when fed to broiler chickens to market age [77]. There was also no impact of an oxidized animal/vegetable fat blend (5 mEq fat/kg diet) on broiler growth performance when fed from 3 to 6 weeks of age [67] and feeding 9 mEq oxidized sunflower oil/kg diet had no impact on growth performance, carcass yield, nutrient digestibility, or markers of lipid peroxidation when fed to broilers from hatch to market age [79].

When using oxidized lipids as a challenge model of oxidative distress in poultry, growth performance may not be the most sensitive or consistent parameter, even if it is the most commercially relevant and reported. In this regard, tissue concentrations and activity of anti- and pro-oxidants or biomarkers of oxidative damage may be better parameters of oxidative distress and antioxidant capacity. For example, feeding broiler chickens increasing concentrations of oxidized poultry fat (0, 3, or 6 mEq fat/kg diet) significantly increased GPX in the red blood cells and the liver [76]. Lipid oxidation biomarkers (TBARS) in the blood and breast muscle tissues were significantly greater in broiler chickens fed 5 mEq oxidized animal/vegetable fat blend/kg diet [67] or in the thigh muscle of broilers fed oxidized poultry fat [77]. Similarly, breast muscle TBARS (day 7 post-storage) were significantly increased and liver and serum vitamin E and A were significantly decreased in broilers fed 7 mEq oxidized soy oil/kg diet [69]. Others have also reported feeding oxidized rapeseed and soy oil resulted in an increase in plasma TBARS and a decrease in plasma, breast muscle, thigh muscle, liver, heart, and fat α-tocopherol [68]. In addition, feeding increasing concentrations of oxidized soy oil significantly increased serum corticosterone of young broilers, confirming the birds were under stress, even in the absence of any impacts on growth performance [78]. Furthermore, serum MDA significantly increased and total antioxidant capacity (T-AOC) and SOD activity in the liver were significantly decreased as the concentration of oxidized soy oil increased in the diet [78]. The effects of oxidized soybean oil were also apparent in the small intestine, with a significant decrease in SOD activity in the ileal mucosa and CAT mRNA expression in the ileum tissue [78]. Laying hens fed oxidized vegetable oil had increased MDA in the plasma and liver, increased DNA damage in the liver, and increased plasma ROS [72]. The ovaries of laying hens are also affected by oxidized oil with an increase in follicle apoptosis and ovary MDA content reported in hens fed oxidized corn oil and corn gluten meal [73].

In the previously mentioned poultry trials, feeding oxidized lipids induced an oxidative distress response in the tissues (blood, liver, muscles or small intestine) in the absence of any, or very limited, impacts on broiler growth performance. Employing oxidized lipids as an oxidative distress challenge model for broiler chickens appears to be an effective inducer of metabolic oxidative distress. The impact of oxidized lipids on growth performance in poultry is inconsistent and this may be related to the type and concentration of oxidized lipid used in the challenge models, the parameter used to measure the amount of lipid oxidation [54,80] and feed intake of broiler chickens, which does not appear to be negatively influenced by rancid or low-quality fat [54,78]. Evaluation of challenge models, additives, and/or methods to alleviate oxidative distress should include tissue markers of oxidative distress and antioxidant status, as well as growth performance and livability. The most responsive tissue biomarkers appear to be the expression of antioxidant enzymes (SOD, CAT, GPX, T-AOC), markers of lipid peroxidation (TBARS or MDA) as well as a measure of fat-soluble vitamins A and E of which vitamin E is an important part of the oxidant-defense system and greatly affected by oxidized lipids.

- Swine

In swine, weaning is a key period involving different stress components (nutritional, environmental and social changes) which affect the oxidative and redox status of piglets [81]. In this regard, weaning provides an interesting model per se of oxidative distress but with large individual variation [82,83]. Consequently, application of a dietary oxidative distress factor may help to standardize in vivo experiments. Inclusion of peroxidized fat in pigs diets from soybean oil [84,85,86], fish oil [87], corn oil [84,85,86] and DDGS oil [88] has been shown to decrease growth performance in piglets and growing pigs suggesting therefore to be a good model (Table 2).

The detrimental effect on animal performance when pigs are fed with oxidized soybean oil, one of the most common lipid sources in pig diets, can be explained by a slight decrease in energy digestibility but also due the metabolic oxidative distress in the pigs measured by the oxidative damage and antioxidant capacity. Rosero et al. (2015) demonstrated that lipid peroxidation (6% soybean oil exposed to heat 80 °C during different days) diminished growth, function and morphology of the small intestine, decreased digestion of energy and lipids in nursery pigs, increased MDA concentrations and reduced the T-AOC in the jejunal mucosa [92]. Comparable results were reported by Silva-Guillen et al. (2020) when feeding 6% peroxidized soybean oil to nursery pigs. The authors reported decreased growth, T-AOC, serum vitamin E and MDA and increased protein carbonyl [91]. In addition, Kerr et al. (2020) reported that feeding 10% thermally oxidized soybean oil at different temperatures and times, to weaned piglets increased oxidative distress as measured by markers of lipid damage (serum and urinary F2-ISP concentration and urinary ISP excretion) but had no effect on energy, lipid, N digestibility or intestinal permeability [89].

Chang et al. (2019) used peroxidized corn oil (5% inclusion to weaned piglets) in commercial conditions and reported negative effects on FCR and an increase in the number of pigs removed for medical treatment, pigs culled for low end weight and mortality. T-AOC and vitamin E concentrations measured in the serum decreased, but concentrations of MDA, 8-OHdG and protein carbonyl were not affected by peroxidation [86]. Similarly, Hanson et al. (2016) reported that the inclusion of 6% peroxidized corn oil to nursery pig diets reduced growth performance and serum vitamin E concentration, with no impact on selenium or TBARS [85]. Boler et al. (2012) showed that inclusion of peroxidized corn oil in finishing barrows impaired growth performance and decreased the antioxidant enzyme GPX and concentration of vitamin E in the liver [84]. In sows, Su et al. (2017) reported that feeding oxidized corn oil from day 85 of gestation to day 21 of lactation, did not markedly affect reproductive performance but decreased serum activities of total SOD and Mn-SOD across lactation and increased placental gene expression of GPX, CAT and SOD [93]. Additionally, peroxidized oil from animal origin, such as fish oil, also showed a depression on the growth performance of piglets, decreased protein digestibility, decreased activities of antioxidant enzymes (SOD and GPX) and increased concentration of MDA in plasma and liver [87].

The previous results could indicate that inclusion of thermally oxidized lipids, independently of the origin, in swine diets has a detrimental effect on the metabolic oxidative status of the animals measured by oxidative damage and specific endogenous antioxidants. In addition, vitamin E concentration in plasma or serum seems to decrease consistently in animals fed peroxidized lipids and could be considered a good indicator of metabolic oxidative status in pigs. Thus, quantification of peroxidation level of lipid sources for swine is critically important to design quality control programs for oil and fat sources and to increase profitability of pork production, especially for weaned pigs that are expected to be the most vulnerable to poor lipid quality.

- Fish

Oxidized oil has been shown to induce gastrointestinal and systemic oxidative distress in multiple freshwater and marine fish species of commercial interest including, Atlantic salmon (Salmo salar) [62,94], rainbow trout (Oncorhynchus mykiss) [95,96], Arctic char (Salvelinus alpinus) [97], Atlantic halibut (Hippoglossus hippoglossus) [98,99], gilthead sea bream (Sparus aurata) [98,100], turbot (Scophthalmus maximus) [98], Siberian sturgeon (Acipenser baeri) [101], African catfish (Clarias gariepinus) [102], carp (Cyprinus carpio) [60,103], Nile tilapia (Oreochromis niloticus) [97], largemouth bass (Micropterus salmoides) [104,105], Wuchang bream (Megalobrama amblycephala) [106] and the Amur minnow (Rhynchocypris lagowski) [107]. These studies show that consumption of oxidized fish oil is often not only associated with impaired gastrointestinal function but also causes apoptosis, inflammation, skeletal deformities, reduced performance and in the worst of cases impacts survival (Table 3).

Fish tissues and their feeds typically contain high concentrations of essential long-chain n−3 PUFAs, particularly docosahexaenoic acid (DHA) and eicosapentaenoic acid (EPA). In the presence of oxygen, PUFAs are susceptible to oxidation, particularly in the event of poor feed manufacture or inappropriate storage conditions. Inherently susceptible to oxidation due to a high degree of unsaturation, PUFAs require protection from degradation by antioxidants, both in fish feeds and in vivo. The stabilization of unsaturated lipids in fishmeal and fish feeds now relies on one less effective and widely used antioxidant due to the ban of ethoxyquin in April 2020 following the EU-issued, “Decision 2017/962 on controlling Ethoxyquin”, in 2017.

Both lipid hydroperoxides and secondary carbonyl compounds in oxidized fish oils are absorbed by fish and in the worst of cases can impair metabolism and cause pathological effects including muscular dystrophy [60,103]. It is suggested that in fish, at least a portion of hydroperoxides are absorbed into multiple organs after decomposition in the intestinal tissue to carbonyl compounds [60]. Oxidized oils have been reported to reduce the uptake of total lipids suggesting an effect of lipid degradation products on energy allocation [96,97,104]. The composition of lipids retained in the whole body and liver are suggested to reflect the oxidation products ingested from the diet. In salmonids and gadids, consumption of oxidized oils results in higher levels of saturated and monounsaturated fatty acids than reduced levels of unsaturated counterparts [62,96,99]. The differential uptake of various oxidation products may, however, depend on the species [98].

Oxidized lipids have been shown to induce antioxidant defense responses both enterically and systemically. The majority of studies on the effects of oxidized oils in fish have reported the modulation of key endogenous antioxidant enzymes including CAT, SOD, GPX and glutathione reductase (GSR). Whilst not confirmed by protein activity, increases in RNA suggest the antioxidant response to oxidized oils in fish is likely modulated by the Keap1-Nrf2-ARE pathway [96,107], which is conserved in fish [52]. The responses of endogenous antioxidant enzyme activities to oxidized oils are mixed across studies, with reports of both up- and down-regulation depending on the species. Consistent with the dietary absorption of the degradation products of oxidized oils, the modulation of both activity and transcript abundance of antioxidant enzymes has been reported in intestinal tissue [107], blood plasma [105,106], liver [97,98,100,105,107], muscle [107], and the whole body [95,96,101]. Non-enzymatic antioxidants are also likely to play an important role in the maintenance of redox balance in fish fed oxidized oils, as decreases of plasma, liver and whole-body tocopherol and retinol have been reported [95,96,104]. These changes in non-enzymatic antioxidants and endogenous enzymes are also reflected by decreases in T-AOC in plasma, liver and muscle, across fish species [105,107].

The feeding of dietary oxidized oils can also result in oxidative damage to the intestinal tract and other organs. Two commonly used markers of lipid peroxidation in fish are, 8-ISP produced by the radical-catalyzed peroxidation of arachidonic acid, and MDA determined by TBARS. These have both been widely reported to increase in the intestine, plasma, liver, muscle and whole body of fish fed oxidized oils. In salmon, increased MDA present in the digesta of fish fed dietary oxidized oils may be absorbed increasing plasma MDA [94]. Increased plasma MDA may, however, also result from the conversion of absorbed hydroperoxides to MDA in the intestinal tissue. The presence of MDA in the muscle indicates muscular oxidative distress and can explain the muscular dystrophy reported in several fish species fed oxidized oils [60,108]. The susceptibility to oxidative damage is likely to depend on the species [98]. Some studies report that levels of MDA are more pronounced in the liver than the whole body, which may be due to the significant hepatic storage of vitamin E [98]. However, in some cases, oxidative damage in the liver from dietary oxidized oil is associated with decreased tocopherol and degenerative hepatic pathologies [104]. Oxidative damage in the intestinal tract is associated with (1) an impaired brush border ultrastructure revealed by shortened, disorganized and loosely arranged villi, (2) inflammation and activation of PI3K-Akt/NF-κB/TCR signaling, and (3) increased plasma cortisol [106]. Numerous studies report that depending on the lipid oxidation level in the diets, measured oxidative distress can often be associated with reduced growth and feed utilization [60,96,98,101,102,106,107]. However, oxidized oil-induced oxidative distress has also been shown to occur without effects on growth [95,99,100]. Conversely, some studies in tilapia and largemouth bass even reported improved growth [97,104]. The reason for improved growth arising from dietary oxidized oils has yet to be elucidated. The type of oxidation product may have some influence on growth, as in Arctic char, effects on growth are only observed with primary oxidation products and not secondary oxidation products [97].

The severity of oxidative effects induced by dietary oxidized lipids depends not only on the level of oil oxidation, but also on the life stage of fish and the quantity of oils required in the diets of different species. Most studies have focused on juvenile fish, which are more susceptible to dietary oxidative distress due to a lack of a complete development of the endogenous antioxidant defense system and/or a delayed response of this system [96,98]. Endogenous antioxidant enzyme defenses (GPX, CAT, SOD) are present in early life stages but at lower levels of activity [95]. In early life stages, levels of non-enzymatic antioxidants including vitamins A and E are present in tissues at higher levels than in later life where they play a key role in antioxidant defenses against the damaging effects of oxidized oils [95,101]. Tilapia have been shown to be more tolerant to oxidized lipids than char, which may, in part, be due to the lower levels of lipid required for tilapia (6%) than char (20%) diets [97].

Oxidized oils may be used to model the utility of functional additives for the amelioration of gastrointestinal and systemic oxidative distress. Functional nutrients, particularly vitamins, have been shown to reduce the effects of oxidized oils in fish diets [108]. Vitamin E has been demonstrated to reduce or even abrogate the effects of oxidized oils in halibut [99], sea bream [98,100] and catfish [102]. A combination of vitamin E and selenium reduced effects of oxidized oils in largemouth bass [104] and other species [108]. Some studies report a lack of effect of vitamin E, so the benefit of vitamin E may depend on the species [98]. Vitamin A has also been shown to diminish the detrimental effects of oxidized oils in Siberian sturgeon [101]. Dietary oxidized oils, which are relatively simply produced by the exposure of fish oils to heat (40–70 °C), and/or air or oxygen effectively induce postprandial oxidative distress in the gastrointestinal tract and systemically. The systemic effects are possibly due to the absorption of lipid degradation products causing oxidative distress in multiple organs. The effects seen in the muscle indicate that filet quality may also be impacted by oxidized oils.

### 4.2. Nutritional Imbalances

Other means by which ROS production can be increased in the gastrointestinal tract include amino acid deficiencies and chronic exposure and/or excess supplementation of heavy metals or proteins (Table 4).

#### 4.2.1. Heavy Metals

Metal ions are essential for basic cellular processes and form the catalytic centers of many critical enzymes. However, high concentrations of some metals can induce ROS production and can also interfere with endogenous antioxidants [120].

Copper is an essential micronutrient included in trace mineral premixes to meet the nutritional requirements of the animal. Broilers have a minimum copper requirement of 15–16 mg/kg feed [121,122] whereas laying hens have a minimum copper requirement of 8 mg/kg feed [123]. Copper is often supplemented at concentrations above requirement (125 to 250 mg/kg diet) in piglet diets, due to its growth promoting properties [124]. Copper is also an important co-factor involved in many intracellular enzymatic functions, including the antioxidant enzyme CuZn-SOD. However, copper can act as a pro-oxidant in diets and in the body because copper ions are actively oxidized and reduced and may catalyze the formation of hydroxyl radicals, which can lead to lipid peroxidation [125]. Chronic exposure to excess copper can induce oxidative damage, liver cirrhosis and damage to the brain, small intestine and other organs. Oxidative damage from copper toxicity is primarily associated with the ability of free copper ions to participate in the formation of ROS, particularly the hydroxyl radical from H_2_O_2_ [126].

The two major Cu sources used in the swine industry, CuSO4 and tribasic CuCl (TBCC; Cu hydroxychloride), differ greatly in their chemical characteristics and can result in different physiological responses. In some studies, feeding weanling pigs with 225 mg Cu/kg diet from CuSO4 or TBCC showed greater lipid peroxidation (based on MDA concentrations) and less liver GSH concentrations with CuSO4 than control or TBCC pigs [116,117]. Fry et al. (2012) suggested that the covalent bonding of Cu to the hydroxyl groups in TBCC allowed Cu to gradually become soluble in the small intestine, thus resulting in less oxidative distress in the intestine than CuSO4.

Arsenic (As), a semi-metal element in nature, is commonly found in water, soil, food, and other materials in both organic and inorganic forms. Arsenic is mainly distributed through the environment by water cycling and is toxic even at low concentrations in the water or feed. Arsenic trioxide (As_2_O_3_) may interact with intracellular ROS, preventing the natural antioxidant systems from re-balancing oxidative distress, leading to cell damage. Feeding Hy-line chickens 300 mg CuSO_4_/kg feed alone or in combination with 30 mg As_2_O_3_/kg feed for 4, 8 or 12 weeks significantly decreased anti-hydroxyl radical (AHR), CAT and GPX and significantly increased MDA levels in the duodenum, jejunum and ileum [109]. Similarly, feeding 25 or 30 mg As_2_O_3_/kg feed for 30, 60 or 90 days significantly decreased CAT, GSH, and GPX in the spleen, bursa, and thymus, and increased MDA concentration in the same tissues [111]. Ajuwon et al. (2011) fed meat-type chickens 250 mg CuSO_4_/kg feed from 3 to 6 weeks post-hatch and reported a significant decrease in SOD, CAT and GSH and significant increases in MDA concentration in the blood and liver [110]. Furthermore, the intestinal tissues of chickens fed excess CuSO_4_ and As_2_O_3_ for 12 weeks showed signs of necrosis, cellular apoptosis, mucosal surface erosion and inflammatory lymphocyte infiltration [109]. Heat shock protein and inflammatory cytokine mRNA levels also increased in the small intestine, in a time-dependent manner, in the chickens fed CuSO_4_ and/or As_2_O_3_ [109]. These results confirm chronic exposure to excess concentrations of CuSO_4_ and/or As_2_O_3_ induce intestinal oxidative distress in poultry through a reduction of the antioxidant defense enzymes. This resulted in an increase in lipid oxidation and cellular necrosis. The increase in biomarkers of lipid oxidation and cell damage, including programed cell death, were more apparent as supplementation time increased and at greater concentrations of As_2_O_3_. Therefore, the use of CuSO_4_ or As_2_O_3_ as a challenge model to induce oxidative distress within the gastrointestinal tract might require dose and time-dependent modifications depending on the production status and age of the animal at the start of the experiment.

Manganese acts as a cofactor of the antioxidant enzyme, mitochondrial Mn-SOD, and may influence the antioxidant capacity in the organism by protecting cells against damages caused by ROS and hydro- or lipid peroxides. However, depending on the concentration, the impact of antioxidant molecules may shift from antioxidative to prooxidative [127] and therefore supplementing feed with trace elements is a balancing act. In the European Union, for most species and categories, maximum dietary concentrations of 150 mg Mn/kg feed have been defined based on animals performance, health, environmental aspects as well as consumer safety [128]. Schwarz et al. (2017) compared two levels of inorganic Mn inclusion (20 vs. 150 mg/kg Mn DM) in pigs from 31 to 116 kg and reported that high Mn diets impaired average daily gain, increased CAT activity in liver, GPX in plasma and T-AOC in muscle, whereas it decreased CAT activity in plasma [119]. Those results indicate that feeding Mn up to the maximum legal level cannot be recommended.

In addition to Mn, iron and zinc can also influence the antioxidant capacity in pigs. Pu et al. (2016) investigated the effects of dietary trace mineral (Cu, Fe, Mn and Zn) supplemental strategies in pigs from 6.5 and 115 kg by feeding NRC requirements or a commercial trace mineral supplement, which provided higher concentrations of minerals (e.g., for 6.5–12 kg BW: Cu 6 vs. 170 mg/kg; Fe 100 vs. 120 mg/kg; Zn 100 vs. 120 mg/kg; Mn 4 vs. 30 mg/kg, respectively). The results showed TBARS, protein carbonylation, and 8-hydroxyguanine (8-OHG) concentrations were significantly increased in pigs fed the commercial trace mineral supplement compared with those fed the NRC requirements diet. These results indicate that long-term dietary mineral exposure with the commercial supplement level could cause hepatic oxidative damage in pigs [118].

#### 4.2.2. Protein and Amino Acids Levels

- Protein

SBM supplemented to fish diets is associated with pathological effects on the gastrointestinal tract including oxidative distress. With its high protein content, attractive cost and availability, SBM is one of the most widely used plant-based ingredients to reduce reliance on marine-derived aqua feed ingredients. Dietary inclusion of SBM is widely known to cause effects on gut health and associated microbiota due its content of antinutritional factors, such as saponins, which can cause pathological effects on gut morphology and inflammation, particularly in carnivorous fish (reviewed in [129,130]). Fishmeal replacement by SBM in aqua feeds is typically between 20 and 40%, at which enteritis can be detected in both omnivorous and carnivorous marine and freshwater fish [129]. Several studies have shown that oxidative distress is also associated with SBM inclusion in sea bream [131], turbot [132,133], Siberian Sturgeon [134], yellow catfish (Pelteobagrus fulvidraco) [135], grass carp (Ctenopharyngodon idella) [136], large yellow croaker (Larimichthys crocea) [137] and totoaba (Totoaba macdonaldi) [138]. SBM contains high levels of carbohydrates, which are particularly challenging for carnivorous fish, but can be somewhat ameliorated by using SBM concentrates (Table 5).

SBM induces gastrointestinal oxidative distress, particularly at the mid and distal segments, and is often associated with inflammation, reduced digestive capacity and impaired intestinal structure. In catfish, an increase of SBM inclusion from 20% to 36% results in intestinal oxidative distress marked by modulated antioxidant enzymes including glutathione-S-transferase (GST), CAT and SOD. In addition, the scavenging of superoxide anion and hydroxyl radical are decreased and levels of MDA and protein carbonyl are increased. This oxidative imbalance is alleviated by supplementation of lysine and methionine indicating the low levels of these amino acids in SBM, may be one factor causing SBM-induced oxidative distress [135]. In yellow croaker, increasing SBM from 12% to 31% results in decreased digestive capacity, impaired uptake of P, Mn, and Zn, decreased SOD and T-AOC, and a concomitant increase in MDA. These effects are ameliorated by the addition of 1.6% citric acid to the diet [137]. Due to its role in transcription factors and as a cofactor of Cu/Zn-SOD, the restoration of Zn uptake may contribute to the restoration of oxidative balance [139]. Similarly, 1.5 to 3% citric acid also alleviates SBM-induced oxidative distress in the distal intestine of turbot. This study also showed the restoration of the distal gastrointestinal structure, mucin expression, and the return of microbiota diversity to that similar of fish fed a control fishmeal diet containing no SBM [132].

Glycinin and β-conglycinin, which constitute 65–80% of the protein fraction of soybeans, are known to cause gastrointestinal oxidative distress in fish. In carp, T-AOC, SOD, CAT, GST, GSR, and GSH are all down regulated by glycinin, particularly in the mid and distal intestine. MDA and protein carbonyls are also increased by glycinin but only in the mid and distal intestine, not the proximal intestine. Interestingly, dietary glycinin is shown to have an inhibitory effect on the translocation of Nrf2 to the nucleus, but only in the mid and distal intestine, which partially explains the decreased activity of endogenous antioxidant defense enzymes in these intestinal segments. Dietary glycinin is also associated with upregulated NADPH oxidase, a potent generator of superoxide radicals, typically generated by innate immune cells [135,136]. Interestingly in mice, rather than being produced by the presence of immune cells, enterocytes and not myeloid cells are the primary producers of superoxide generated by NADPH oxidases [140]. In these studies, the amino acid glutamine, with its strong anti-inflammatory properties, was found to restore the oxidative status of the gastrointestinal tract, as previously suggested by Ding et al. (2016) in cobia (Rachycentron canadum) [141].

Gastrointestinal oxidative distress induced by dietary inclusion of SBM may not remain localized but can “spill-over” throughout the body of fish and causing systemic oxidative distress. Inclusion of 58% SBM in sea bream is associated with the infiltration of eosinophils in the distal intestine and a marked upregulation of CSF-1R, a high-affinity tyrosine-kinase receptor that mediates the effects of the cytokine CSF-1 and a potent marker of macrophage activation. Concurrently, hepatic activity of antioxidant enzymes including GST, GPX, CAT, SOD and GSR are increased and associated with an unexplained lipid accumulation in hepatocytes [131]. In turbot, addition of 35% SBM to diets also causes intestinal inflammation, marked by a decreased villi height, reduced expression of the anti-inflammatory cytokine TGF-β (transforming growth factor-β) and increased expression of the pro-inflammatory cytokines TNF-α (tumor necrosis factor-α), IL-1β (interleukin-1β), and IL-8 (interleukin-8). This inflammatory response is concomitant with hepatic oxidative distress as indicated by a downregulation of SOD, GPX and peroxiredoxin 6 (PRX6), and an increase of MDA in the liver. Resveratrol, a natural polyphenol compound with antioxidant properties was effective in partially reversing these effects both in the intestine and liver [133]. Similarly, in totoaba, whilst effects in the gastrointestinal tract were not examined, 39% inclusion of SBM concentrate significantly increased hepatic MDA [138]. The process of the spread of SBM-induced oxidative distress in fish may be as that of inflammatory bowel disease (IBD) in humans. In human IBD, the increased oxidation in the intestinal wall is mirrored within the systemic circulation, as oxidized thiol groups extend deep into the extracellular space of the intestinal wall and the extracellular space, which is a conduit for the systemic transport of oxidized thiols throughout the body. As blood proteins, including albumin, contain 60–75% of the total circulating pool of thiols, blood constituents are intimately linked to the global extracellular redox state [142].

As in fish, SBM is also the most widely used plant protein source in pig diets and the immunologically active soybean proteins, glycinin and β-conglycinin, cause delayed hypersensitivity and localized immune responses in pigs [143]. Alternative protein sources, bioprocessing, or fermentation of SBM has been shown to reduce antinutritional factors, allowing SBM to be fed to nursery pigs without adversely affecting performance [144,145]. Ma et al. (2019) evaluated the effects of partially replacing (12%) SBM soy protein concentrate, fermented SBM, or fish meal with enzyme-treated SBM in weaned pigs during the first two weeks after weaning [146]. They observed that after the two weeks piglets fed with all the alternatives to SBM had increased BWG and improved FCR. Furthermore, piglets fed the fish meal and enzymatic treated SBM had higher activities of SOD, GPX and T-AOC, and lower MDA concentration at the serum, indicating that the alternative protein ingredients could play an important role alleviating oxidative distress and lowering the degree of lipid oxidative injury in weaned pigs. Moreover, the negative impact of SBM on oxidative distress has been also explored longer than the post-weaning period in pigs. Schwerin et al. (2002) concluded that pigs fed soybean protein compared to casein had increased oxidative distress responses in the liver, measured by an increase in upregulating GST, peptide methionine sulfoxide reductase, apolipoprotein A-I, organic anion transport polypeptide 2, calnexin and heat shock transcription factor 1 genes [147].

In swine, especially in weaned piglets, both source and level of dietary protein are known to influence enteric health. The effects of dietary protein sources on growth and gut health of pigs have been studied extensively, but there is still a paucity of information on the effect of dietary protein sources on the oxidative status. In addition to the type of protein source used in weaned piglet diets, the level of protein inclusion and the feeding pattern employed might have an impact on gastrointestinal functionality and oxidative status. Wu et al. (2016) evaluated the effects of different dietary protein levels (LP, low protein 15.7%; C, “normal protein” 17.0%; HP, high protein 18.3%) and daily feeding pattern (twice per day, C-C; LP-HP; HP-LP) on weaned piglets for 42 days. The authors reported no significant effect on growth performance. However, piglets fed the HP diets had an increase in serum SOD concentration, in the morning, compared to piglets fed the control group. There was no significant effect of protein levels or the feeding patterns on T-AOC, CAT or MDA levels in the serum [148]. Perez Calvo et al. (2019) evaluated the effect of different dietary protein levels from SBM (LP, low protein 18.2% vs. HP, high protein 24.6%) and different feeding patterns (ad libitum LP vs. feed restriction HP) on weaned piglets during two weeks after weaning. Results demonstrated no effect on growth performance. Oxidative distress index in the plasma increased when pigs were fed with the HP diet [149]. Additionally, Bacou et al. (2019) found in the same study, an upregulation of the oxidative distress genes (NOX, GPX, SOD, CAT and peroxiredoxin) in the liver and Peyer patches [150].

- Amino acids

Methionine is the first limiting amino acid for the growth of chickens. Methionine is essential for protein synthesis, immune function and more than half of the ingested methionine acts as a precursor of cysteine, which is required for GSH synthesis. In addition, methionine is a scavenger and readily reacts with ROS such as hydroxyl radicals or H_2_O_2_. Methionine deficiencies result in a decrease of antioxidant capacity in the small intestine, liver and plasma of meat-type chickens. For example, Tao et al. (2018) and Wu et al. (2018) reported significant decreases in SOD, CAT, GSH and GPX, or the ability to inhibit hydroxyl radicals in the intestinal tract or cecal tonsils of broilers fed 52% to 70% of their methionine requirement from hatch to market age. In the same experiments, lipid peroxidation (measured as MDA) was significantly increased as well as cell apoptosis in the small intestine [114] or cecal tonsils [115]. The impact of methionine deficiency on markers of oxidative distress was only significant after birds were fed the deficient diets for approximately 3 weeks, possibly indicating exhaustion of the antioxidant capacity over time. This exhaustion eventually led to significant decreases in the antioxidant enzymes and increases in cell damage and lipid peroxidation. Unfortunately, growth performance and mortality were not reported in the previously mentioned trials. The impact and commercial applicability of methionine deficiencies as challenge models of oxidative distress and evaluation of antioxidants to alleviate oxidative distress remains to be determined. Supplementation of methionine to methionine-deficient diets significantly improved the antioxidant (GSH, GPX, GSSG, GSH:GSSG) properties in the liver, serum and small intestine of meat-type chickens [112]. Chen et al. (2013) fed broilers total methionine at 99% or 119% of their requirement and reported birds fed 119% of their methionine requirement had enhanced antioxidant capacity as measured by significant increases in serum SOD and liver GSH:GSSG ratios. This resulted in significant decreases in liver MDA or GPX when broilers were fed 119% of their methionine requirement as compared to those fed 99% of their requirement [113]. Due to the importance of methionine in antioxidant function, deficiencies or excess supplementation of dietary methionine may provide a good nutritional model of oxidative distress. Furthermore, the use of nutritionally adequate diets which are then supplemented with excess methionine might mitigate any confounding effects associated with methionine deficiencies, such as reduced gain and meat yield, increased feed intake and fat deposition [151] and allow interpretation of any results solely on oxidative distress and antioxidant functionality. However, there appears to be a limit to the benefits of excess dietary methionine supplementation, and an over-supply (3× the requirement) can be considered toxic, resulting in reduced growth rate and increased feed efficiency [152]. Therefore, as with Cu and As supplementation, the dose and time to exhaust the animal’s antioxidant capacity should be considered when implementing one of these challenge models of oxidative distress.

### 4.3. Mycotoxins

Mycotoxins are secondary fungal metabolites often found as contaminants in agricultural commodities all over the world which, when ingested, exhibit various cytotoxic, mutagenic, and carcinogenic effects on humans and animals, and consequently significant economic losses to livestock farms. More than 400 different mycotoxins have been isolated and chemically characterized. Those of major medical and agricultural concern are aflatoxins, fumonisins, ochratoxins (OTA), citrinin, trichothecenes (DON, T-2 toxin, nivalenol (NIV), zearalenone (ZEA), and patulin (PAT) [153]. Several aspects of the intracellular action of mycotoxins have been elucidated and the induction of oxidative distress and ROS have become one of the major triggers of lesions commonly associated with mycotoxin exposure, as reviewed by Silva et al. (2018) [154]. The toxic effects of the mycotoxins depend on the type and amount of exposure, which varies inter-species and even inter-individuals and relies on nutrient and physiological factors. Table 6 contains a summary of various examples of mycotoxin challenge models, including the dose and the effect on performance and oxidative distress parameters on poultry, swine and fish.

- Poultry

The liver is considered the principal target organ for aflatoxicosis. Oxidative distress is reported to play an important role in the pathologies associated with aflatoxicosis through influences on the Nrf2 signaling pathway. The Nrf2 pathway protects cells from oxidative damage. Rajput et al. (2019) reported that 1 mg aflatoxin B1 (AFB1)/kg feed down-regulated Nrf2 genes and target proteins (HO-1, GPX, NQO1, GCLC) in the liver of broiler chickens [167]. Balogh et al. (2019) fed broiler chickens diets contaminated with 149 μg AFB1/kg of feed and reported significant decreases in the expression of GSSG in the liver and GSH in the plasma. The concentration of AFB1 was 7.5 times the EU limit and also resulted in significant decreases in feed intake, weight gain and liver weight [165]. However, other biomarkers, such as plasma or liver TBARS, GPX, GSH, or GSR were not influenced by AFB1. This may suggest the birds were able to mount an adequate antioxidant response and effectively protect lipid membranes from oxidative damage due to the short duration of the trial (2-weeks).

The impact of OTA on markers of oxidative distress in poultry is limited. Zhai et al. (2020) fed 2 mg OTA/kg to ducks from hatch to day 21 and reported decreased liver CAT only. There was no effect of OTA on T-AOC, SOD, MDA, GPX, or mRNA expression of antioxidant genes (HMOX-1 or Nrf2) [169]. The lipid peroxidation marker MDA was increased in lymphocytes from 45-day old broiler chickens exposed to graded levels of OTA (0.001 to 1 μg OTA/mL) [186].

Numerous experiments in broilers or ducks highlight the negative and significant impact of fusarium toxins-including DON, T-2, fumonisin B1 (FB1) and/or combinations with ZEA or OTA on biomarkers of lipid peroxidation and antioxidant enzymes in the liver, spleen, plasma and small intestinal tissues. In most experiments, growth performance was not measured in conjunction with markers of oxidative distress. However, where it was measured, FCR was increased by 11 points [159], feed intake or BWG were decreased [157,161] or there was no effect of FB1 alone or in combination with DON on growth of broilers [156,164]. In contrast, the impact of T-2 toxin on markers of oxidative distress was measured only in livers [158] or splenocytes [160]. In both tissues, T-2 toxin significantly increased ROS and MDA and decreased expression of SOD, CAT, GPX suggesting T-2 has a profound impact on markers of oxidative distress in the liver or spleen.

When considering the impact of fusarium toxins on biomarkers of oxidative distress in broiler chickens, FB1 appeared to have the greatest impact in the plasma, liver, spleen, or small intestine, regardless of its feed concentration. FB1 significantly increased serum H_2_O_2_, markers of lipid peroxidation (MDA/TBARS), protein oxidation products (carbonyl), and/or nitrite/nitrate level [157,161,164] whereas serum SOD and GPX were decreased [157]. Furthermore, tissue concentrations of MDA, CAT, ascorbic acid, HMOX, ROS, H_2_O_2_, MDA, carbonyl, GST, and/or nitrite/nitrate level were significantly increased in the liver or ileum [156,157,159,161,164]. Interestingly, SOD, GPX, and in some cases GST were not influenced or significantly decreased by FB1 contamination of broiler diets [157,159,161]. The differentially expressed biomarkers and antioxidant enzymes, for example CAT, SOD, the increase in plasma H_2_O_2_, may indicate FB1 induces oxidative distress through the production of H_2_O_2_, which is a substrate for CAT and an end product of SOD activity. In this case, the reduction of SOD and increase CAT may be a protective mechanism to limit the amount of H_2_O_2_ produced as a result of FB1 toxicity. Further work to characterize these findings is warranted in broilers.

Awad et al. (2014) reported no impact of DON toxin on plasma, heart, kidney, liver or duodenum markers of lipid peroxidation (MDA/TBARS) in meat-type chickens [162]. However, blood lymphocyte DNA damage [162] and MDA [186] and MDA/TBARS in the jejunum were significantly increased by DON [162,168]. The impact of DON on markers of oxidative distress in poultry is apparent, but less clear or consistent compared with FB1 or T-2 toxin. This is further exacerbated by testing the combination of DON with other mycotoxins, such as ZEA [163,170], T-2 [166], or FB1 [156]. Osselaere et al. (2013) observed that DON has a significant impact on tight junction proteins in the small intestine, resulting in an increase in cell permeability. Therefore, the effects of DON, measured as markers of oxidative distress, may be secondary to the inflammatory response associated with increased intestinal permeability and loss of tight junction functionality [155] in poultry tissues.

- Swine

Mycotoxins contamination levels in pig feedstuffs are usually not high enough to cause an overt disease but may result in economical loss through changes in growth, production, and immunosuppression. In Europe, regulation and/or recommendations exist for 6 mycotoxins that may be present in pig feed: aflatoxins, ochratoxins, fumonisins, ZEA, and trichothecenes, principally DON, T-2 and HT-2 toxins [187]. Thus, the current review focuses on the studies using those mycotoxins as challenge model and their effect on the oxidative distress biomarkers.

Aflatoxins are hepatotoxic and carcinogenic, they also they also display immunotoxic properties impairing both the innate and acquired immune response. Mechanistically, AFB1 is associated with excessive ROS production which causes oxidative distress and leads to amino acid damage via metal-catalyzed oxidation with the inhibition of cellular 20S proteasomes, a proteolytic complex that degrades oxidized proteins. AFB1 inhibits also Nrf2/ARE signal pathway and increases that of the nucleotide excision repair (NER pathway) resulting in DNA and lipid damage and affecting the cellular defense [188]. In pigs, Taranu et al. (2020) used 320 mg AFB1/kg of feed in weaned piglets diet as a challenge model and reported an increase in both 8-OHG and 8-OHdG levels as well as in protein carbonyl content in the liver relative to control diet (by 42.7% and 43.0%, respectively) [178].

OTA is a secondary metabolite produced by fungi of *Aspergillus* and P*enicillium* genera. OTA is mainly nephrotoxic but can also cause hepatotoxicity, mutagenicity, teratogenicity, neurotoxicity and immunotoxicity. Pigs are generally considered to be the most sensitive species to the nephrotoxicity induced by OTA and following oral ingestion, around 66% of OTA is rapidly absorbed and metabolized in intestine, liver and kidney. OTA alters intestinal barrier and absorption functions, thus facilitating the translocation of the toxin from gut lumen into the bloodstream and, both urine and feces are important excretory pathways for the toxin what might entail a cyclical re-contamination. Exposure to low levels of OTA (0.5 mg/kg or 0.025 mg/kg) can decrease pig growth rate and feed efficiency with or without the reduction of the feed consumption and alter serum chemical chemistry as indicator of kidney damage in piglets [180] or growing finishing pigs [181]. The impact of OTA on markers of oxidative distress in pigs has not been largely reported. Marin et al. (2017) studied the effect of a sub-chronic exposure (30 days) to 0.05 mg/kg OTA and reported a decreased in gene expression of both markers of signaling pathways involved in inflammation (NF-kB and eNOS in duodenum and of iNOS in the colon, while no effect for kidney) and inflammatory cytokines (IL-6, IL-8, IL-12, IL-17A, IL-18) in the colon more than in kidney of the piglets exposed to toxin. The expression of HO-1 gene, an important marker of oxidative distress was not affected by the exposure to OTA in the duodenum, colon, or kidney. However, contaminated feed induced a slight increase of SOD activity in both the kidney and the duodenum, with no effect on CAT or GPX activities or on T-AOC in any of the analyzed tissues [179].

FB1 is reported to cause diseases such as porcine pulmonary edema. Moreover, when fed to pigs, FB1 reduces viability and promotes lactate dehydrogenase release in porcine renal epithelial cells, suggesting induction of nephrotoxicity [189]. FB1 is also reported to affect the porcine immune system by inhibiting lymphocyte proliferation and controlling the Th1/Th2 cytokine balance [190]. Another study reported decreased expression of IL-8 in the gut of pigs following the oral administration of 0.5 mg/kg FB1, although other cytokines were unaffected but no specific oxidative distress parameters were evaluated [191].

ZEA is a nonsteroidal estrogenic mycotoxin that cause diseases related to infertility in pigs, especially pre-pubescent gilts. Studies have shown that ZEA is quickly absorbed after oral administration to pigs and humans and is reduced to α- and β-zearalenol via metabolism in intestinal cells; consequently, intestinal epithelial cells might be exposed to toxic substances at various concentrations. It was also found that chronic intake of a ZEA-contaminated diet can change the structure of intestinal villi and reduce the expression of tight junction proteins. In fact, in addition to its estrogen-like effects, ZEA and its metabolites trigger lipid oxidation in several cell lines, indicating that it has an important effect on oxidative distress. It has been reported that ZEA (2.77 mg/kg) significantly increased MDA concentrations and GPX activity and decreased the SOD activity in the jejunum of sows [192]. Further, ZEA supplemented at 0.1, 0.5, 1.0, or 1.5 mg/kg to post-weaning gilts showed that the activity of total SOD and GPX decreased linearly and quadratically and that MDA content increased quadratically in the ileum and mesenteric lymph nodes with increasing ZEA in the diet [173]. Additionally, the expression of Nrf2 and GPX1 immunoreactive proteins in the ileum and mesenteric lymph nodes were significantly enhanced with increasing ZEA. Similarly, diets containing 1 mg ZEA/kg of feed in weaned piglets diet affecting the oxidative status, increasing MDA and decreasing SOD, T-AOC and GPX in the liver. However, the addition of 150 mg/kg feed of vitamin C decreased the negative effects of ZEA on the liver, ZEA residues and oxidative distress. This resulted in a decrease in MDA and an increase in the levels of SOD, T-AOC and GPX in the liver of piglets, confirming that vitamin C can alleviate damage to the liver of weaning piglets by modulating the nuclear receptor signaling pathway [172].

DON, belongs to the Trichothecene mycotoxins and can cause growth performance decreases, immune dysfunction or organ injury to humans and animals after being ingested and seriously affects their health. It is colloquially known as “vomitoxin” due to its emetic effects in piglets and thus swine are more susceptible to DON, compared to other domesticated animals, probably due to the metabolism by their gut microbes [193]. DON is absorbed in the upper part of the small intestine by passive diffusion and significantly affects the intestinal morphology and structure of piglets, causing intestinal mucosal damage, including villus atrophy, crypt cell necrosis, intestinal cell metabolic disorder, and changes in protein biosynthesis Many studies have found that the early symptoms of DON exposure to pigs are related to decreased growth performance [175,176,194,195]. Moreover, pigs fed DON-contaminated diet (4 mg/kg) have shown to inhibit activities of SOD, GPX, GSH, and T-AOC [175]. Another recent study reported DON exposure caused a decrease of SOD in the jejunum and GPX in the liver and increased the concentration of MDA in the serum and H_2_O_2_ level in the liver. In addition, DON induced intestinal inflammation, impaired the intestinal barrier, and disturbed the gut microbiota homeostasis [176]. Similarly, Holanda and Kim (2020) reported that DON (1.2 vs. 3.2 mg/kg) mildly compromised growth performance and increased the oxidative distress of pigs, as observed by an increase in MDA and GSH concentrations in jejunal mucosa. However, no differences were observed in nutrient digestibility or histological parameters [174].

Pigs are generally exposed to two or more types of mycotoxins simultaneously, rather than a single mycotoxin. Different combinations of mycotoxins have a synergistic effect on environmental toxicity, while some combinations cause antagonistic effects. Several review papers have described the interaction effects caused by different types of mycotoxins [196]. This section will cover some of the studies in pigs including mycotoxins combinations. Frankic et al. (2008) evaluated the effect of DON and T-2 toxin inclusion in weaned piglet diets and they observed that body weight and FCR were impaired. DON significantly increased the amount of DNA damage in lymphocytes by 28%. Moreover, the levels of T-AOC were lowered by addition of DON. T-2 toxin significantly impaired daily live weight gain and feed conversion, increased the amount of DNA damage in lymphocytes by 27%, decreased total serum IgG and did not alter plasma T-AOC. However, plasma and 24-h urinary MDA excretion rate and erythrocyte GPX levels did not differ among the groups [171]. On the other hand, when LeThanh et al. (2016) evaluated the effect of the inclusion of DON and ZEA at 3.1 and 1.8 mg/kg, respectively, in weaned piglets diets, they did not observe a negative effect on growth performance but an increase in MDA plasma levels and SOD activity in liver [177].

- Fish

Whilst over 300 mycotoxins have been identified [197] five types are regularly found in aquaculture feeds including aflatoxins, fumonisins, ochratoxins, trichothecenes and ZEA [198]. These fungal toxins are typically heat stable and resistant to destruction during the extrusion of aqua feeds [199]. The levels and types of mycotoxins in commercial fish feeds therefore tend to reflect the contamination of the raw ingredients [200]. The increasing use of plant-based ingredients in aqua feeds, is one of the factors leading to an increased awareness of mycotoxins in the aquaculture industry [199].

The contamination of aqua feeds with mycotoxins is dependent on the geographical region and its climate, the seasons and the raw ingredients. Mycotoxins such as aflatoxins and ochratoxins, can arise during inadequate feed or ingredient storage, whilst others arise in the growing crop. For example, in the field soybean and corn crops are not typically contaminated with aflatoxin, but are more likely contaminated with DON, fumonisins and/or ZEA [201]. Mycotoxin analysis of commercial compound fish feeds from Europe and Asia shows that the occurrence and level of contamination is typically higher in tropical and sub-tropical regions, such as southeast Asia, than in the more temperate climates of northern regions of Europe and the Americas [201]. Fungi, and hence mycotoxins, arise in aqua feeds due to several reasons including, improper storage conditions in high temperature and/or humidity, poor manufacturing, for example insufficient drying time or a lack of preservatives or anti-molds, and the selection of ingredients, which can carry fungal spores that are resistant to extrusion [199].

Mycotoxins are known to cause toxicity to several fish species in both experimental and commercial settings, the main health burden being related to chronic mycotoxin toxicity. In this respect the most studied mycotoxin to date in fish is AFB1. The severity of mycotoxicosis depends on the type of mycotoxin, dose and duration of exposure, lifecycle stage, sex, dietary status, interaction with other toxic compounds and health status. Fry are more sensitive to aflatoxins than older fish. Exposure to mycotoxins can involve multiple types of toxins. A wide body of literature documents the detrimental effects of mycotoxins in fish, recently reviewed by Anater et al. (2016) [202]. These include decreases in growth, hepatic pathologies and immune dysfunction. Warm-water fish such as Nile tilapia (Oreochromis niloticus) or channel catfish (Ictalurus punctatus) are generally more resistant to mycotoxins (aflatoxins) than cold-water species, particularly salmonids. Marine species are also sensitive to aflatoxins including red drum (Sciaenops ocellatus) and sea bass (Dicentrarchus labrax) [198].

At practically relevant concentrations, mycotoxins cause oxidative distress in fish, particularly in the liver where these toxins are metabolized. Studies including an assessment of the oxidative effects of mycotoxins have largely been limited to tilapia and the effects AFB1 or T-2. Depending on the study and the dose of aflatoxin, mycotoxin-induced oxidative distress can be associated with decreased growth and often hepatic pathologies. In SE Asia where tilapia are extensively raised, the average and maximum level of aflatoxin in commercial aqua feeds is 50 and 220 µg/kg, respectively [201]. Even within these ranges found in commercial feeds, increases in hepatic MDA have been observed as early as two weeks of experimental feeding with diets containing 16 µg/kg AFB1. Interestingly, this is despite a lack of significant changes in SOD, GPX or CAT. In this study, hepatic oxidative distress was associated with significant effects on zootechnical and health parameters, including liver pathologies [203]. Further increases in dietary AFB1 cause changes in endogenous antioxidant enzymes. In tilapia fed either 20 or 100 µg/kg AFB1 for 12 weeks, hepatic GPX and GST gene expression were increased by the higher level of AFB1. These changes in endogenous antioxidant enzymes were associated with reduced growth, impaired FCR, and liver damage marked by a decreased liver somatic index (LSI) and increased serum aspartate aminotransferase (AST), alanine aminotransferase (ALT) and alkaline phosphatase (ALP) activities [204]. Similarly, in tilapia fed 200 µg/kg AFB1 for one month, phagocytic activity, total blood proteins and catalase activity were all reduced with a concurrent increase in ALT, creatinine and MDA [182]. The detoxification of AFB1 per se is one factor driving mycotoxin induced oxidative distress. In tilapia fed 200 µg/kg AFB1 for 12 weeks, expression of the hepatic detoxification enzyme cytochrome P450 1A, (CYP1A) was increased in conjunction with a down regulation of SOD gene expression [204]. Conversely, the activity of liver CYP1A protein, which is required for the detoxification of AFB1 to AFB1-8,9-epoxide (AFBO) was decreased in tilapia fed diets with 85 µg/kg AFB1 for 20 weeks. The detoxification of AFBO also occurs in liver and consumes GSH explaining AFB1-induced oxidative distress [183].

The oxidative effects of higher experimental doses of aflatoxins affects several fish species and can cause oxidative distress beyond the liver, the effects of which can be ameliorated by the addition of antioxidant ingredients. Diets containing 2500 µg/kg AFB1 fed to tilapia for 30 days resulted in elevated hepatic AST, ALT, and ALP activity. This was associated with reduced activities of GSH, GPX and CAT in the gill, liver and kidney which was also accompanied by an increased MDA and in these tissues. These effects were ameliorated with fucoidan, a complex polysaccharide found in brown seaweed that enhanced serum biochemical and tissue antioxidant responses [205]. Similarly, the effects of 200 µg/kg AFB1 on tilapia reported by Abdel et al. (2017) were reduced by dietary inclusion of fennel essential oil, which contains tanethole, limonene, fenchone and vitamin C as major components that confer hepatoprotective antioxidant effects [205]. In addition to lipid peroxidation, aflatoxins also cause DNA and protein oxidation. 3000 µg/kg AFB1 fed to tilapia for 84 days caused reduced SOD and CAT activity and an increased hepatic MDA which was associated with liver pathologies. Consistent with oxidative distress, this study also showed the oxidative effects of AFB1 on DNA, as determined by an increased tail moment as measured by the comet assay [206]. In silver catfish (Rhamdia quelen) fed diets with 1177 µg/kg AFB1 lipid peroxidation and protein carbonylation levels in the brain were increased after 21 days. In this study the burden on antioxidant defenses was shown by an associated decrease in serum and hepatic antioxidant capacity, vitamin C levels, and activities of GPX and CAT [207].

T-2 toxin, a trichothecene mycotoxin produced by Fusarium species also causes oxidative distress and has been detected in 79.5% of feeds sampled from China, at concentrations ranging from 10 to 735 μg/kg [184]. In tilapia, 24 mg/kg T-2 toxin in diets fed for 20 days causes hepatic oxidative distress associated with histopathological changes including increases in GST activity. Similarly, in carp (Cyprinus carpio) exposed to 5.3 mg/kg dietary T-2 for 28 days hepatic CAT and GST, were modulated with a concurrent increase in TBARS, whereas GPX and GSR were not affected. In addition to the liver, in the posterior kidney, CAT and GPX were also modulated together with an increase in TBARS [185].

## 5. Conclusions

The dietary inclusion of oxidized fats and oils, SBM, heavy metals, amino acids, proteins, or mycotoxins may be used as effective models of oxidative distress in poultry, swine and fish. These dietary ingredients, toxins, or formulation modifications were selected as models due to the (1) commercial relevance in production animal diets, (2) simplicity to add to diets, and (3) reported influences on biomarkers of gastrointestinal oxidative distress, which can also spread throughout the entire organism to cause systemic oxidative distress and affect multiple organs including the liver and muscle. These challenge models and measurements of biomarkers will allow for studies to test the effects of multiple compound classes that improve the effects of postprandial oxidative distress caused by challenging feed ingredients, nutrient concentrations or toxins.

In poultry, feeding peroxidized lipids consistently influenced biomarkers of oxidative distress in the tissues, particularly antioxidant enzymes, fat soluble vitamins, and markers of lipid peroxidation. However, this did not always manifest as a significant impact on growth performance or feed efficiency, possibly due to the lack of effects of peroxidized oil on feed intake. Chronic exposure to high concentrations of CuSO_4_ and/or As_2_O_3_ induced intestinal oxidative distress in poultry by reducing the antioxidant defense enzymes. This resulted in an increase in biomarkers of lipid oxidation and cellular necrosis. The impact of heavy metals on oxidative distress appeared to be time and dose dependent and this should be considered when excess exposure of CuSO_4_ or As_2_O_3_ are employed as challenge models of oxidative distress. Similarly, the use of deficient or surplus essential amino acids resulted in changes in biomarkers of oxidative distress in a time-dependent manner, indicating a depletion of the animals’ redox system or T-AOC over time. Finally, the impact of mycotoxins on oxidative distress in poultry depends on the mycotoxin and contamination dose fed. The fusarium toxins, FB1 and T-2 appeared to induce the greatest impact on growth performance and/or tissue markers of oxidative distress in broiler chickens or laying hens. An interesting observation in broilers exposed to FB1 was the increase in plasma H_2_O_2_, in conjunction with a reduction of SOD activity and an increase of CAT activity. This response highlights the animals attempt to limit the amount of H_2_O_2_ produced as a result of FB1 toxicity and suggests FB1 may be a good model to induce H_2_O_2_-derived oxidative distress in poultry.

In swine, dietary inclusion of peroxidized fat (independently of the origin), high levels of SBM as protein source, high levels of heavy metals as Cu or Mn and mycotoxins, have shown to have a negative impact on growth performance in pigs, especially in weaned piglets. Several studies have evaluated the effect of those challenges models on digestibility, intestinal integrity and immune status but there is limited information regarding the effects on the oxidative status of the animals. However, when oxidative stress biomarkers have been measured, inconsistent responses on enzymatic, non-enzymatic or oxidative damage (lipid, protein and DNA) have been observed even when a similar challenge is applied what reveals the complexity of the redox homeostasis. Nevertheless, it seems that markers of lipid damage as TBARS or MDA are the most commonly used and show an effect independently of the challenge model. Assessment of vitamin E and T-AOC are good indicator of oxidative stress when inclusion of peroxidized fat in feed and endogenous antioxidant enzymes when mycotoxins (ZEA and DON) are present in feed.

In fish, the dietary inclusion of either oxidized fish oils or SBM may be used to effectively model a production-relevant oxidative distress in multiple fish species. Both dietary ingredients are relatively simple to add to diets and cause gastrointestinal oxidative distress, which can also spread throughout the entire organism to cause systemic oxidative distress affecting multiple organs including the liver and the muscle. Several studies have effectively tested the effects of multiple compound classes that ameliorate the effects of postprandial oxidative distress caused by challenging feed ingredients. Mycotoxins, namely AFB1 and T-2, consistently induce oxidative distress in fish and may also be effective as models of in vivo oxidative distress. The effects mycotoxicosis are particularly realized in the liver due to its role in detoxifying mycotoxins absorbed from the diet. These effects are observed even at doses experienced by fish in the field. Dietary antioxidants have been shown experimentally to ameliorate the oxidative effects of mycotoxins, which in their metabolism have been shown to generate oxidizing molecules and consume reducing agents, such as glutathione. Further studies are required to assess the potential effects of other mycotoxins identified in aqua feeds, notably in cold-water and marine species, which are particularly susceptible to these fugal toxins. Diets may be formulated with contaminated raw ingredients or purified preparations of mycotoxins, which due to their stability would be expected to be well recovered in finished feeds.

## Figures and Tables

**Table 1:** Summary of the dietary oxidative stress challenges models using oxidized fat in poultry, including type of fat, oxidation conditions, resultant fat characterization and inclusion level in feed, and results including growth performance impact, oxidative stress markers evaluated classified by enzymatic, non-enzymatic or oxidative damage.

Animal (Age)	Type of Fat Source (Inclusion Level)	Oxidation Conditions	PV, mEq/kg	Growth Performance	Oxidative Distress Biomarkers			Ref.
					Enzymatic	Non Enzymatic	Damage	
Broiler chicken (28–42 d)	Fat blend (5%)	RT until a PV of 100	100	No effect			Increased TBARS in blood and breast muscle	[67]
Broiler chicken (10–38 d)	9% rapeseed oil 2% soyabean oil	15 mg/kg Fe^2+^, 7.5 mg/kg Cu^2+^, 660 mg/kg H_2_O_2_, stirring	156	Tendency to decrease live weight	No effect on GPX in liver	Vit E decreased in plasma, breast and thigh muscle, liver, heart and fat	Increased plasma TBARS	[68]
Broiler chicken (1–39 d)	Soyabean oil (3% S, 4% GF)	Heating, 95 °C, continuous bubbling air,	180	Decreased BW, gain, and FCR		Decreased serum and liver vitamin A and E	No effect plasma TBARS	[69]
Broiler chicken (1–21 d)	Soyabean oil (5%)	Heating, 90 °C, continuous bubbling,	244	No effect on gain, decreased intake, increased FCR	Decreased liver GPX, no effect plasma GPX, increased liver GPX, GSR	Increased plasma, liver T-AOC	Decreased liver TBARS, no effect plasma TBARS	[70]
Laying hens (40–44 wks)	Soyabean oil (1, 2, and 4%)	Air at RT, 3 months, heated 185 °C, cooled, heated	21, 56, and 88	Decreased egg production, increased FCR, no effect on intake				[71]
Laying hens (53–59 wks)	Sunflower oil (2%)	Intermittent heating, 185 °C, air	29.2	Decreased FI, no other effect		Decreased plasma glutathione	Increased plasma, liver MDA, increased liver DNA damage, increased hepatic carbonyl, liver ROS	[72]
Laying hen (38–46 wks)	corn oil (2%) CGM (10%)	Heating 100 °C	corn oil, 7.6 CGM, 40	Corn oil = decreased egg production, DFI, FCR and egg weight; CGM = decreased egg production and DFI		Tendency to decrease T-AOC	Increased pre-hierarchical follicle apoptosis, ovary MDA,	[73]
Broiler chicken (1–49 d)	Fat blend (3% S; 6% GF)	Heating at 135–140°F	0, 75, 150	Decreased BWG, increased FCR				[74]
Broiler chicken (1–49 d)	Fat blend (3% S; 6% GF)	Heating at 135–140°F	0, 75, 150					[75]
Broiler chicken (1–21 d)	Poultry fat (5.4%)	Heating, 80 °C, aeration, stirring	0, 3, or 6	No effect	Increased GPX in RBC and in liver			[76]
Broiler chicken (10–47 d)	Poultry fat (4%)	Fried, 110–120 °C,	38.7	No effect on gain, improved FCR			Increase TBARS in thigh meat over time (stored chilled)	[77]
Broiler chicken (1–21 d)	Soyabean oil (4%)	Heating, 200 °C	4, 25, 57, or 73	No effect on gain or FCR	Decreased ileum SOD, CAT	Decreased liver T-AOC	Increased liver, jejunum MDA	[78]
Broiler chicken (1–42 d)	Sunflower oil (6%)	O_2_, aerated, heating 70–80 °C	148	No effect	Decreased plasma SOD, no effect plasma GPX		No effect plasma MDA	[79]

S = starter diet; GF = grower/finisher diet; CGM = corn gluten meal; RT, room temperature; PV, peroxide value; BWG = Body weight gain; FCR = feed conversion ratio; DFI = Daily feed intake; SOD = superoxide dismutase; CAT = catalase; GPX = glutathione peroxidase; GSR = glutathione reductase; MDA = malondialdehyde, TBARS = thiobarbituric acid reactive substances; T-AOC = total antioxidant capacity; RBC = red blood cells.

**Table 2:** Summary of the dietary oxidative challenges models based on oxidized fat discussed within this review in swine, including type of fat, oxidation conditions, resultant fat characterization and inclusion level in feed, and results, including growth performance impact, oxidative stress markers evaluated classified by enzymatic, non-enzymatic or oxidative damage.

Animal Phase (Age/BW)	Type of Fat Source and Inclusion Level	Oxidation Conditions	PV	Growth Performance Effect	Oxidative Distress Biomarkers			Ref.
					Enzymatic	Non Enzymatic	Damage	
G pigs (80 kg)	Corn oil (5%)	Heating 95 °C and bubbling air	150 mEq/kg	Decreased BWG	Decreased GPX	Decreased vit E in liver and	No effect on TBARS nor free carbonyl at plasma	[84]
Weaned piglets (28 d)	Corn oil (3, 6 or 9%)	Heating at 185 °C during 12 h with force air flow	5.7 meq O_2_/kg	Decreased BWG		Decreased vit E in serum but no effect on Se levels at serum.	No effect on TBARS at serum.	[85]
Weaned piglets	Corn oil (5%)	Heating at 65° for 12 d with forcing air	163 mEq/kg oil	Increased FCR		Decreased T-AOC and vitamin E at serum.	No effect on MDA, PC, nor 8-OH-2dG at serum.	[86]
Weaned piglets (28 d)	Fish oil (5%)	Addition of FeSO4⋅7H_2_O, CuSO4⋅5H_2_O, H_2_O_2_ and water, and heated for 60 h at 37 °C.	786.50 meq O_2_/kg	Decreased BWG	Decreased SOD, GPX		Increased MDA in plasma and liver	[87]
Weaned piglets (6.6 kg)	DDGS (30%)	From ethanol commercial plant	84.1 mEq/kg	Decreased BWG		Increased vit E in loin muscle	No effect on TBARS in loin muscle	[88]
Weaned piglets (28 d)	Soybean oil (SO; 10%)	Heating at 45 °C for 288 h, 90 °C for 72 h, or 180 °C for 6 h	11.5, 19.1, and 13.4 mEq/kg feed	Peroxidized SO at 90 °C reduced ADG ADFI			ISP increased at serum and urine but no effect on TBARS, 8-OH-2dG at serum, urine nor liver	[89]
G pigs (25 kg)	Soybean oil (10%)	Heating 45 °C for 288 h, 90 °C for 72 h, or 180 °C for 6 h);	96, 145, and 4.0 mEq/kg,	Decreased BWG and increase FCR				[90]
Weaned piglets (21 d)	Soybean oil (6%)	Heating 80 °C for 12 h and continous bubble air		Decreased BWG, FI and increased FCR		T-AOC and Vit E in serum decreased;	Decreased MDA and PC increased but no effect on 8-OH-2dG	[91]
Weaned piglets (21 d)	Soybean oil (6%)	Heating (80 °C) and constant oxygen flow for 0, 6, 9and 12 d		Reduced FI and BWG		Decreased T-AOC	Increased MDA in the jejunal mucosa	[92]
Sows (85 d gestation–d21 lactation)	Corn oil (2%)	Heating at 95°C for 72 h and bubbling air	250 mEq/kg	No effect on reproductive performance but decreased feed intake during lactation	Decreased SOD and Mn-SOD at serum across lactation but no effect on GPx	No effect on vit E nor C	No effect on MDA	[93]

PV = peroxide value; BWG = Body weight gain; FCR = feed conversion ratio; DFI = Daily feed intake; SOD = superoxide dismutase; CAT = catalase; GPX = glutathione peroxidase; GSR = glutathione reductase; MDA = malondialdehyde, TBARS = thiobarbituric acid reactive substances; T-AOC = total antioxidant capacity, ISP = F2-isoprostanes; PC = protein carbonyls, 8-OH-2dG = 8-hydroxy-2′-deoxyguanosine.

**Table 3:** Summary of the dietary oxidative challenges models based on oxidized fat discussed within this review in fish, including type of fat, oxidation conditions, resultant fat characterization and inclusion level in feed, and results, including growth performance impact, oxidative stress markers evaluated classified by enzymatic, non-enzymatic or oxidative damage.

Animal Phase (Age/BW)	Type of Fat Source and Inclusion Level	Oxidation Conditions	PV	Growth Performance Effect	Oxidative Distress Biomarkers			Ref.
					Enzymatic	Non Enzymatic	Damage	
Atlantic salmon (67g)	Oxidized herring oil	Aerating at room temperature for 1 month	200 mEq/kg	No effect			Increased TBARS in liver, fillet and large intestine content	[62]
Rainbow trout (1.5 g)	Oxidized salmon oil (8%)	Bubbling air into oil for 90 h at 50 °C	234 mEq/kg,	No effect	Increased SOD, CAT, GPX gene expression			[95]
Rainbow trout (66 g and 1.5 g)	Oxidized fish oil (12%)	Bubbling air into oil for 6 days at 50 °C	93 and 144 mEq/kg	Reduced wet weight	Increased whole boy GST, CAT, GR and GPX gene expressions	Reduced whole body Vitamin E	Increased TBARS and ISP	[96]
Artic charr (2.5 g) Tilapia (1.7 g)	Oxidized herring oil (152.7 g/kg; charr and 76.2 g/kg tilapia)	Bubbling with pure oxygen at 50–70 °C for 2–7 days	183, 56, 33 mEq/kg (Charr) 447 and 33 mEq/kg (Tilapia)	Decreased FBW (highest level oxidized oil; charr) Increased FBW (Tilapia)	Increased hepatic CAT and GPX activities and decreased SOD activities (Charr)			[97]
Sea bream, Turbot and Halibot	Oxidised anchoui/Sardine oil (12.6%)	Bubbling air into oil for 24 h at 50 °C	42 mEq/kg	Decreased FBW and SGR (all species)	Increased hepatic SOD, CAT and GPX activities (Sea bream), hepatic GST and GR acitivities (Turbot) and hepatic SOD, GST and GSR activities (Halibot)		No effect (Sea bream) Increased hepatic TBARS and ISP (Turbot/Halibot)	[98]
Sea bream (1.2 g)	Oxidized tuna orbital oil (12.6%)	Heating for 24 h at 50 °C in oxygen-rich atmosphere	42 mEq/kg	No effect	Increased SOD and CAT activities and reduced GPX activities (30 days) Decreased CAT and GPX activities (60 days)	Increased GSH (60 days)	Increased hepatic MDA (30 and 60 days) and ISP (60 days)	[100]
Sturgeon (0.4 g)	Oxidized capelin oil (4 and 8%)	Bubbling air into oil for 48 h at 50 °C	245 mEq/kg	Reduced growth	Increased whole body SOD and CAT activities		Increased TBARS and ISP	[101]
African catfish (15 g)	Blender cod-live/corn oil (1:1; 60 g/kg)	Aeration for 30 days at 22 °C		Decreased FBW		Decreased vitamin E in muscle, liver, plasma, heart and spleen		[102]
Large amount bass (31.5 g)	Oxidized fish oil (70 g/kg)	Heating at 50 °C with continous air injections	155, 275 and 574 mEq/kg	Increased FBW and WG with lowest level oxidized oil	Increased hepatic SOD and GPX activities with highest dose of oxidized oil	Increased hepatic T-AOC with highest dose of oxidized oil	Increase hepatic MDA with highest dose of oxidized oil	[105]
Blunt snout bream (5.2 g)	Oxidized salmon oil (2; 4 and 6%)	Heating at 37 °C for 14 days with air injections	8, 16 and 23 mmol/kg	Decreased FBW, SGR and FCR in dise response manner	Increased SOD and GPX with increased oxidized oil	Decreased GSH with increasing oxidized oil	Increased MDA with increasing oxidized oil	[106]
Rhynchocypris agowski Dybowski (4.5 g)	Oxidized fish oil (4%)	Heating at 70 °C	107, 195, 293 and 403 mEq/kg	Reduced SGR and BWG (highest levels of oxidized oil)	Decreased GPX, CAT and SOD in dose response manner in liver and muscle	Decreased GSH and T-AOC in liver, intestinal tract and muscle in dose response manner	Increased hepactic and intestinal MDA	[107]

PV = peroxide value; FBW = Final body weight; WG = weight gain; SGR = specific growth rate; SOD = superoxide dismutase; CAT = catalase; GPX = glutathione peroxidase; GSR = glutathione reductase; GSH = glutathione; GST = Glutathione-S-Transferase; MDA = malondialdehyde, TBARS = thiobarbituric acid reactive substances, ISP = isoprostanes; 4-HNE = 4-hydroxyalkenals; T-AOC = total antioxidant capacity.

**Table 4:** Summary of the dietary oxidative challenge models using minerals imbalances in poultry and swine diets, including nutrient level, and results, including effect on growth performance and markers of oxidative distress classified as enzymatic, non-enzymatic, or damage.

Animal (Age/BW)	Nutrient (Concentration)	Growth Performance	Oxidative Distress Biomarkers			Ref.
			Enzymatic	Non Enzymatic	Damage	
Male pullets	CuSO_4_ (300 mg/kg feed), As_2_O_3_ (30 mg/kg feed)		Decreased small intestine CAT, GPX		Decreased small intestine AHR (anti-hydroxyl radical), increased small intestine MDA	[109]
Broiler chicken (21–42 d)	CuSO_4_ (250 mg/kg feed)		Decreased blood, liver SOD, CAT	Decreased blood, liver GSH	Increased liver, blood MDA	[110]
Male pullets (0–90 d)	As_2_O_3_ (0, 7.5, 15, 30 mg/kg feed)		Decrease thymus, bursa, spleen CAT, GPX	Decreased thymus, bursa, spleen GSH	Increased thymus, bursa, spleen MDA	[111]
Broiler chicken (0–21 d)	Met (0.05 or 0.25%)	0.25% Met increased BW, daily gain, improved FCR	0.25% Met increased GPX in serum	0.25% Met increased liver GSSG, decreased liver, small intestine GSH:GSSG, decreased small intestine GSH, GSSG		[112]
Broiler chicken (0–21 d)	Met (99 or 119% of requirement)	119% Met improved gain, FCR in light weight chicks	119% Met increased serum SOD, increased liver GPX	119% Met increased liver GSH:GSSG	119% Met decreased liver MDA	[113]
Broiler chicken (0–42 d)	Met (52 or 70% of requirement)		Decreased small intestine SOD, CAT, GPX	Decreased small intestine ability to inhibit hydroxyl radicals, decreased small intestine GSH	Increased small intestine MDA	[114]
Broiler chicken (0–42 d)	Met (52 or 70% of requirement)		Decreased cecal tonsil SOD, CAT, GPX,	Decreased cecal tonsil ability to inhibit hydroxyl radicals, decreased cecal tonsil GSH	Increased cecal tonsil MDA	[115]
Weaned piglets (21 d)	225 mg Cu/kg dietCuSO_4_ vs. TBCC	No effect after 33 days of supplementation independently of Cu form		Decreased total liver GSH		[116]
Weaned piglets (21 d)	225 mg Cu/kg dietCuSO_4_ vs. TBCC	No effect			Increased duodenal MDA	[117]
Growing pigs (6 5–115 kg)	Cu, Fe, Mn, Zn at NRC vs. commercial trace mineral supplement				Commercial trace mineral supplement increased TBARS, PC, and 8-OH-2dG	[118]
Growing pigs (31–16 kg)	Mn (20 vs. 150 mg/kg)	Decreased in BWG	Increased GPX and CAT in plasma and CAT in liver	Increase T-AOC in muscle	No effect TBARS in muscle	[119]

Met, methionine; BWG = Body weight gain; FCR = feed conversion ratio; DFI = Daily feed intake; SOD = superoxide dismutase; CAT = catalase; GPX = glutathione peroxidase; GSR = glutathione reductase; MDA = malondialdehyde, TBARS = thiobarbituric acid reactive substances; T-AOC = total antioxidant capacity; PC = protein carbonyls, 8-OH-2dG = 8-hydroxy-2′-deoxyguanosine.

**Table 5:** Summary of the dietary oxidative challenges models using soybean meal in fish diets, including inclusion level and results, including effect on zootechnicalperformance and markers of oxidative distress classified as enzymatic, non-enzymatic, or damage.

Animal (Age/BW)	Protein (Inclusion Level)	Growth Performance	Oxidative Distress Biomarkers			Ref.
			Enzymatic	Non Enzymatic	Damage	
Turbot (9.6 g)	SBM (37.9%)	Decreased FBW, SGR and FI	Decreased intestine SOD and GPX gene expression	Decreased intestinal T-AOC	Increased intestinal MDA	[132]
Turbot (7.5 g)	SBM (35.10%)	Decreased FBW and SGR	Decreased hepatic SOD activity			[133]
Yellow catfish (17 g)	SBM (36%)	Decreased FBW and increased FCR	Decreased intestine CAT activity	Decreased intestine GSH	Increased intestine MDA and protein carbonyl	[135]
Large yellow croaker (7.7 g)	SBM (30.6%)	Decreased FBW and SGR	Decreased intestine SOD activity	Decreased intestinal T-AOC	Increased intestinal MDA	[137]
Totoaba (7.5 g)	SBM (19.5 and 38.9%)	Reduced growth (SBM 38.9%)	Increased SOD activity (SBM 19.5%)		Increased hepatic MDA	[138]

FBW = Final body weight gain; SGR = Specific growth rate; FI = feed intake; SOD = superoxide dismutase; GPX = glutathione peroxidase; MDA = malondialdehyde; T-AOC = total antioxidant capacity.

**Table 6:** Summary of the dietary oxidative challenges models using mycotoxins in poultry, swine and fish diets, including nutrient and concentration and results, including effect on growth performance and markers of oxidative stress classified as enzymatic, non-enzymatic, or damage.

Animal Phase (Age/BW)	Mycotoxin and Level	Growth Performance	Oxidative Distress Biomarkers			Ref.
			Enzymatic	Non Enzymatic	Damage	
Broiler chickens (10–31 d)	DON (7.5 mg/kg)				Increased XOR and HMOX in the jejunum	[155]
Broiler chickens (1–15 d)	DON (5 mg/kg) +FB1(20 mg/kg)	No impact on gain or FCR			Increased HMOX in the ileum, and down-regulated XDH in the jejunum	[156]
Broiler chickens (1–20 d)	FB1(10 mg/kg)	Decreased intake and gain; no impact on FCR	Increased liver CAT, no effect on SOD		Increased TBARS in serum but not in liver	[157]
Broiler chickens (1–21 d)	T2 toxin (0, 0.5, 1, 2 mg/kg)		Decreased expression of GPX, CAT, SOD		Increased MDA	[158]
Broiler chickens (1–21 d)	FB1 (100 mg/kg)	Increased FCR	Increased CAT and no effect on SOD	lncreased ascorbic acid	Increased MDA, no effect on non-protein thiols or GST	[159]
Broiler chickens (1–21 d)	T2 toxin (0, 0.5, 1, or 2 mg/kg)		Decreased SOD and CAT	Decreased GSH-Px	Increased MDA	[160]
Broiler chickens (12–21 d)	FB1 (2.5, 5, 10 mg/kg)	Decreased gain, no effect FI	Decreased serum and liver SOD and GPX	Decreased liver and plasma GST		[161]
Broiler chickens (1–35 d)	DON (10 mg/kg)				Increased jejunum TBARS and blood lymphocyte DNA damaged	[162]
Broiler chickens (14–28 d)	DON (3.4 or 8.2 mg/kg) + ZEA (3.4 or 8.3 mg/kg)		Increased GPX in blood and decreased in liver; no impact in duodenal mucosa nor SOD in RBCs	No impact on vitamin E in RBCs	Increased MDA in liver; no impact on MDA in duodenal mucosa or thioredoxin reductase (TrxR) in the liver	[163]
Broiler chickens (1–42 d)	FB1 (200 mg/kg)	No effect			Increased liver ROS, H_2_O_2_, MDA, carbonyl (protein oxidation products)	[164]
Broiler chickens (21–24 d or 21–35 d)	AFB1 (149 ug/kg)	Decreased FI and BWG	No effect on plasma GPX	Decreased plasma GSH	No effect on TBARS at plasma or liver; increased liver gene expression of GPX4 and decreased gene expression of GSSG	[165]
Broiler chickens (21–28 d)	DON (5, 12, 25 mg/kg) + T2 toxin (0.23, 1.2, 2.4 mg/kg)		Increased liver GPX	Increased liver GPX	No impact on liver MDA;	[166]
Broiler chickens (3–28 d)	AFB1 (1 mg/kg)				Decreased liver protein expression of endogenous antioxidant genes (Nrf2, HO-1, GPX1, NQO1, GCLC)	[167]
Broiler chickens (6–14 d)	DON (19.3 mg/kg)			Decreased GSH in the jejunum and ileum	Increased TBARS in the jejunum. No effect on superoxide anion production/pathways in the jejunum/ileum	[168]
Duck (1–21 d)	OTA (2 mg/kg)		Decreased liver CAT; no effect on SOD	No effect liver T-AOC or GPX	No effect liver MDA or mRNA expression of antioxidant genes (HMOX-1, Nrf2)	[169]
Duck (14–47 d)	DON (4.9 mg/kg) +ZEA (0.66 mg/kg)				decrease in liver MDA; increased free sulfhydryl group and reducing power in liver	[170]
Weaned piglets (11.7 kg)	DON (4 mg/kg) and T2 toxin (3 mg/kg)	DON and T2 decreased DGW and increased FCR	No effect on erythrocyte GPX		No effect on plasma and 24-h urinary MDA excretion rate	[171]
Weaned piglets (12.27 kg)	ZEA (1 mg/kg)		Decreased the level of SOD and GPX in the liver	Decreased the level of T-AOC	Increased MDA in the liver	[172]
Weaned gilts (14 kg)	ZEA (0.1; 0.5; 1 and 1.5 mg/kg)		Decreased SOD and GPX in ileum and MLNs		MDA increased quadratically in the ileum and MLNs. mRNA and protein expression of Nrf2, GPX1, NQO1, HMOX1, Gclm, and Gclc increased in the ileum and MLNs	[173]
Weaned piglets (27 d)	DON (1.2 and 3.2 mg/kg)	Decreased ADG	Decreased GPX in jejunal mucosa		Tended to increase MDA in jejunal mucosa	[174]
Weaned piglets (21 d-35 d)	DON (4 mg/kg)	Decreased ADG, ADFI and FCR	Decreased SOD and GPX in serum	Decreased GSH, and T-AOC in serum	Increased MDA in serum	[175]
Weaned piglets (21 d-42 d)	DON (3.6 mg/kg)	Decreased ADG and ADFI	Decreased SOD in jejunum and GPX in liver	Decreased GPX2 expression levels in jejunum	Increased MDA in serum	[176]
Weaned piglets (6 kg)	DON (3.1 mg/kg) + ZEA (1.8 mg/kg)	No effect on growth performance	Increased SOD in liver	No effect GSH in plasma and liver	Increased MDA in plasma	[177]
Weaned piglets (9.1 kg)	AFB1 (320 mg/kg)	Decrease on BW			Increased 8-OHG and 8-OHdG level as well as in PC content	[178]
Weaned piglets (9.8 kg)	OTA (0.05 mg/kg)		Increased SOD in both kidney and duodenum but no effect on CAT and GPX activities		No effect on expression of HMOX1 gene at gut or kidney levels	[179]
Growing pigs (15–50 kg BW)	OTA (0.5 or 2.5 mg/kg)	Decreased BWG, FI and FCR				[180]
GF pigs (40 kg to 170 kg)	OTA (0.025 mg/kg)	Decreased BWG and FCR but not FI				[181]
Nile tilapia (27 g)	AFB1 (200 µg/kg)		Decreased serum CAT activity		Increased MDA in serum	[182]
Nile tilapia (20 g)	AFB1 (1641 µg/k)	Decreased feed intake and FER				[183]
Nile tilapia (15 g)	T-2 (24.3 mg/kg)	Decreased survival rate and weight gain	Increased GST activity in liver			[184]
Common carp (77 g)	T-2 (5.3 mg/kg)		Decreased liver CAT activity and GPX activity in caudal kidney		Increased TBARS in liver and caudal kidney	[185]

AFB1 = Aflatoxin B1; FB1 = Fumonisin B1; OTA = Ochratoxins; DON = Deoxynivalenol; ZEA = zearalenone; BWG = Body weight gain; FCR = feed conversion ratio; DFI = Daily feed intake; FER = Feed efficiency ratio; XOR = Xanthine oxidoreductase; XDH = Xanthine dehydrogenase; SOD = superoxide dismutase; CAT = catalase; GPX = glutathione peroxidase; GSR = glutathione reductase; GST = Glutathione-S-transferase; MDA = malondialdehyde; TBARS = thiobarbituric acid reactive substances; MNLs = Mesenteric lymph nodes; RBC = Redd blood cells; GSSG = glutathione disulfide; Nrf2 = nuclear factor erythroid 2-related factor 2; NQO1 = quinone oxidoreductase 1, HMOX = hemeoxygenase; Gclm = glutamate-cysteine ligase; Gclc = glutamate-cysteine ligase; 8-OHG = 8-hydroxyguanosine; 8-OHdG = 8-oxo-2′-desoxyguanosine, PC = protein carbonyl.
